# Brain structure and clinical profile point to neurodevelopmental factors involved in pedophilic disorder

**DOI:** 10.1111/acps.13273

**Published:** 2021-01-22

**Authors:** Christoph Abé, Roberth Adebahr, Benny Liberg, Christian Mannfolk, Alexander Lebedev, Jonna Eriksson, Niklas Långström, Christoffer Rahm

**Affiliations:** ^1^ Department of Clinical Neuroscience Karolinska Institutet Stockholm Sweden; ^2^ ANOVA Karolinska University Hospital Stockholm Sweden; ^3^ Department of Clinical Sciences (Psychiatry) Umeå University Umeå Sweden; ^4^ Centre for Psychiatry Research Department of Clinical Neuroscience Karolinska Institutet, and Stockholm Health Care Services Stockholm Sweden; ^5^ Psychiatry South Stockholm Sweden; ^6^ Department of Medical Epidemiology and Biostatistics Karolinska Institutet Stockholm Sweden; ^7^ National Board of Health & Welfare Stockholm Sweden

**Keywords:** cerebral cortex, neurodevelopmental disorder, neuroimaging, pedophilic disorder, psychiatric comorbidity

## Abstract

**Objective:**

Pedophilic disorder (PD) is characterized bypersistent, intense sexual attraction to prepubertal children that the individual has acted on, or causes marked distress or interpersonal difficulty. Although prior research suggests that PD has neurodevelopmental underpinnings, the evidence remains sparse. To aid the understanding of etiology and treatment development, we quantified neurobiological and clinical correlates of PD.

**Method:**

We compared 55 self‐referred, help‐seeking, non‐forensic male patients with DSM‐5 PD with 57 age‐matched, healthy male controls (HC) on clinical, neuropsychological, and structural brain imaging measures (cortical thickness and surface area, subcortical and white matter volumes). Structural brain measures were related to markers for aberrant neurodevelopment including IQ, and the 2nd to 4th digit ratio (2D:4D).

**Results:**

PD was associated with psychiatric disorder comorbidity and ADHD and autism spectrum disorder symptoms. PD patients had lower total IQ than HC. PD individuals exhibited cortical surface area abnormalities in regions belonging to the brain's default mode network and showed abnormal volume of white matter underlying those regions. PD subjects had smaller hippocampi and nuclei accumbens than HC. Findings were not related to history of child‐related sexual offending. IQ correlated negatively with global expression of PD‐related brain features and 2D:4D correlated with surface area in PD.

**Conclusions:**

In the largest single‐center study to date, we delineate psychiatric comorbidity, neurobiological and cognitive correlates of PD. Our morphometric findings, their associations with markers of aberrant neurodevelopment, and psychiatric comorbidities suggest that neurodevelopmental mechanisms are involved in PD. The findings may need consideration in future development of clinical management of PD patients.

**Significant outcomes**

- Patients with pedophilic disorder showed symptoms of neurodevelopmental disorders.
- Pedophilic disorder was associated with structural brain abnormalities in the default mode network.
- Brain abnormalities were related to markers of prenatal androgen exposure.

**Limitations**

- The cross‐sectional study design does not allow for inference regarding causality.
- Structural neuroimaging cannot assess the brain regions’ functional role in the development of pedophilic disorder.
- We studied self‐referred individuals with pedophilic disorder. Conclusions about individuals not seeking clinical help should be drawn with caution.

## 1 INTRODUCTION

Pedophilic disorder (PD) is a paraphilic disorder defined by the *Diagnostic and Statistical Manual of Mental Disorders*, *5*th *edition (DSM*‐*5)* ^1^ as a persistent sexual attraction to prepubescent children, where the individual has acted on these sexual urges, or they cause marked distress to the afflicted individual or interpersonal difficulty. Beyond furthering diagnostics, risk assessment, and treatment for individuals with PD, improved knowledge about its neurobiological origins could aid the prevention of child sexual abuse.

Previous studies have linked PD to conditions where neurodevelopmental factors play substantial roles suggesting that neurodevelopmental factors contribute to the development of PD.^2^, ^3^, ^4^ These factors include genetic and environmental influences on the brain, such as prenatal androgens^3^ affecting brain development, cognitive functioning, and disorder development. Thus, presumable brain alterations in PD are proposed to be present since early childhood or emerging adolescence. In line with aberrant neurodevelopment, individuals with PD presented with lower intelligence compared with individuals attracted to adults.^5^ Other PD‐related neuropsychological deficits include lower processing speed together with impaired attention and prefrontal executive functions, such as response inhibition, task switching, and cognitive reasoning.^4^, ^6^, ^7^ Research investigating anthropometric signs linked to in utero conditions suggest associations between PD and minor physical anomalies,^8^ shorter height^9^ and left‐handedness.^4^ Notably, a higher 2D:4D ratio (the ratio between 2nd and 4th digit length), a putative marker for prenatal androgen exposure,^10^ has been associated with paraphilic interest^11^ but not been investigated specifically in PD. Other indications for neurodevelopmental influences originate from studies addressing psychiatric comorbidity.^4^ Gerwinn et al^12^ found an association between pedophilia and symptoms of the neurodevelopmental disorder attention deficit hyperactivity disorder (ADHD). However, links between PD and autism spectrum disorder (ASD) remain unknown.

A handful of previous, small‐scale neuroimaging studies have addressed putative neurobiological underpinnings of PD. These studies indicate cortical abnormalities in various brain regions and volumetric white matter and amygdala alterations pointing toward altered emotional and arousal networks in PD. Despite heuristic value, prior studies report inconsistent results^4^, ^6^, ^13^ and suffer from substantial shortcomings; small sample sizes, inadequate control groups, and limiting *region of interest* approaches. Moreover, the most recent and largest study combined data acquired with multiple brain scanners.^13^ This previous report suggested that brain abnormalities may rather be attributed to sexual offending behavior than to PD (or pedophilic interest) per se.^13^ A recent review argued that neuroimaging in PD is still in its infancy and suggested replication in larger samples while controlling for potential confounding factors; sexual offending, sexual orientation, psychiatric comorbidity, and medication use.^6^ Hence, the contribution of neurodevelopmental mechanisms to PD remains unclear.

### 1.1 Aims of the study

The aim of this single‐center study was to comprehensively assess measures of brain structure, psychiatric morbidity, and psychological function in a consecutive sample of help‐seeking men with DSM‐5 PD and to compare them with healthy controls. Another goal was to investigate the relations between PD‐related structural brain abnormalities and cognitive function (IQ) and 2D:4D ratios, as a marker for prenatal androgen exposure.

## 2 MATERIAL AND METHODS

### 2.1 Participants

We performed this study within the framework of *Pedophilia at Risk* – *Investigations of Treatment and Biomarkers* (PRIOTAB), a clinical trial and research project.^14^ With a case‐control design (PRIOTAB‐CC), we aimed to identify clinically useful biomarkers and risk factors by investigating PD‐related neurobiology using magnetic resonance imaging (MRI), psychiatric assessment, and cognitive testing. The study was conducted at ANOVA, Karolinska University Hospital, Stockholm, Sweden, a multidisciplinary clinic and research center for sexual medicine, andrology, and transgender medicine. Self‐referred, help‐seeking subjects with PD were recruited through PrevenTell, a Swedish national helpline. PD was confirmed using a structured interview based on DSM‐5 criteria. Age‐matched, male healthy controls (HC), screen‐negative for PD, were recruited in the Stockholm catchment area through Karolinska Trial Alliance and adverts on Karolinska Institutet's homepage. More details on screening and recruitment procedures can be found in the supplemental material or elsewhere.^14^ Fifty‐five adult male individuals with PD and 57 HC completed baseline clinical assessments with four homosexual and eight bisexual participants in each group, respectively. A total of 54 PD patients and 50 HC provided MRI data of sufficient quality for the current analyses. All participants provided written informed consent and the study was approved by the Swedish Central Ethical Review Board.

### 2.2 Psychiatric comorbidity and clinical symptoms

The Swedish version of the Mini International Neuropsychiatric Interview (M.I.N.I.) 6.0.0 tapped co‐existing psychiatric disorders.^15^ Using the 1999 WHO Consultation on Child Abuse Prevention guidelines,^16^ participants were also asked whether they had been sexually abused or exploited before age 15 years.

Symptoms indicative of autism spectrum disorder (ASD) were assessed with the abridged version of the Ritvo Autism and Asperger Diagnostic Scale (RAADS‐14), covering mentalizing deficits (7 items), social anxiety (4 items), and sensory reactivity (3 items). We investigated ASD‐related symptoms using two established cut‐off scores. The first threshold (>13) is based on the suggested screening cut‐off, where results ≤13 imply a non‐ASD diagnosis with a negative predictive value of 99%. The stricter cut‐off (>23) is linked to a positive predictive value of 37% of receiving a full ASD diagnosis in unselected general population samples.^17^

ADHD symptoms were assessed with the 18‐item Adult ADHD Self‐Report Scale – Screen (ASRS‐v1.1).^18^ Each item was rated on a 5‐point Likert scale (0 = never; 4 = very often). Indicated ADHD was coded “1” for respondents endorsing 4+ part A items with “sometimes” or “often” (depending on item), and “0” otherwise. The Alcohol Use Disorders Identification Test (AUDIT)^19^ and the Drug Use Disorders Identification Test (DUDIT)^20^ self‐reports were used to screen for substance use disorders. We used the Hypersexual Behavior Inventory (HBI‐19)^21^ self‐report to assess hypersexual behavior, indicated by an HBI score >52.

### 2.3 General cognitive ability

The Swedish version of the Wechsler Adult Intelligence Scale–IV (WAIS‐IV) was used to assess general cognitive ability.^22^ The full‐scale intelligence quotient (IQ) was estimated from subtests of intellectual functioning across four cognitive areas: Verbal Comprehension Index (VCI), Perceptual Reasoning Index (PRI), Working Memory Index (WMI), and the Processing Speed Index (PSI).

### 2.4 Anthropometric measurements

A research nurse measured participants’ height, weight and the lengths of index finger (2nd digit) and ring finger (4th digit) of the right hand for assessment of the 2D:4D digit ratio.^10^ Digits were measured from the basal crease to the tip using a Vernier caliper. Handedness was assessed with the Swedish version of the Edinburgh Handedness Inventory self‐report.^23^

### 2.5 Structural MRI image acquisition and processing

Brain magnetic resonance imaging (MRI) scans were performed at the Karolinska University Hospital, Huddinge, Sweden, using a 3T medical scanner (Siemens Prisma). We obtained measures of cortical volume, thickness, and surface area for each participant from structural T1‐weighted images using the semi‐automated cortical surface reconstruction and parcellation methods provided by FreeSurfer 6.0.^24^, ^25^ FreeSurfer also provided segmentation of subcortical volumes, cortical parcellations defined by the *Desikan* atlas, and segmentation of white matter (WM) volumes of regions underlying these cortical parcellations. These measures were extracted for secondary analyses, including multivariate classification analyses (described below). Details on image acquisition and processing are described in the Supporting Information.

### 2.6 Statistical analyses

#### 2.6.1 Demographic and clinical variables

Group differences in demographic, anthropometric, and clinical measures were assessed with IBM SPSS Statistics version 25. We used *t* tests for normally distributed data (tested by skewness), the Mann–Whitney *U* test for skewed data and *χ* ^2^‐tests for dichotomous variables. We provided Cohen's *d* values as effect size (ES) measures, calculated as standardized mean differences for parametric data, via biserial rank correlations for non‐parametric data, or from frequency distributions for categorical data.

#### 2.6.2 Case‐control differences in structural brain imaging measures

Group differences in cortical brain imaging measures were performed on vertex level. In the main analysis, we tested for the effect of group (PD vs HC; independent variable of interest) on cortical thickness and WM‐surface area (dependent variables), while correcting for age (regressor of no interest). Cortical volume comparisons were performed for completeness. Secondary analyses comparing groups on subcortical volumes and regional WM segmentations were conducted in SPSS v25, using multiple univariate analyses of covariance (ANCOVAs). More details on analytical methods are described in the Supporting Information.

#### 2.6.3 Sensitivity tests (testing for potential confounds on brain imaging findings)

For sensitivity analyses testing for potential confounders, we extracted cortical thickness, surface area, and volume measures averaged over significant clusters obtained in the main analysis. First, we compared these measures between groups to validate our main findings (Table S2). We then tested for potential confounding effects by demographic or clinical variables (e.g., comorbidities, medication use) on group differences by entering them as additional covariates (one at a time) in the statistical model. In additional tests, when fewer than ten participants had a specific comorbidity/medication, we repeated confounder analyses one at a time after having excluded those individuals. More methodological details are provided in the Supporting Information.

#### 2.6.4 Correlations with 2D:4D (interpretational purpose)

To investigate the potential role of prenatal androgens on cortical structure in PD, we tested for associations between 2D:4D and cortical measures on vertex level, as well as subcortical and WM volumes. Details are described in Supporting Information.

#### 2.6.5 Multivariate pattern and classification analysis

To test whether global morphometric brain patterns expressed in PD are linked to clinical and behavioral characteristics, we used partial least squares (PLS) classification^26^ to categorize between PD and HC. Using FreeSurfer parcellated regional cortical, WM, and subcortical volume measures, we computed a single score for each subject that characterized the magnitude of expression of the identified PD‐related abnormality pattern (here referred to as global morphometric PD abnormality expression score). We then tested for associations between global morphometric PD scores and 2D:4D, total IQ, and variables that affected our main outcome measures indicated by sensitivity analyses (see above). Classification analyses were performed in R (see Supporting Information for details).

## 3 RESULTS

### 3.1 Socio‐demographic, sexuality, and psychiatric characteristics

Table 1 presents group comparisons of socio‐demographic and clinical variables.

**TABLE 1:** Socio‐demographic, sexuality, and psychiatric characteristics among male participants with DSM‐5 Pedophilic Disorder (PD) and matched non‐clinical male control subjects (HC)

Baseline characteristic	PD (*n* = 55)	HC (*n* = 57)	Cohen's *d*; *p*‐value^a^
Age, years, range, M (SD)	18–66, 36 (12)	18–64, 36 (12)	−0.05; *p* = 0.804
Height, cm, M (SD)^b^	182 (6)	181 (6)	0.04; *p* = 0.818
Body mass index, M (SD)^b^	25 (5)	24 (3)	0.21; *p* = 0.272
2D:4D ratio, Mdn (IQR)^c^	0.96 (0.07)	0.95 (0.06)	0.34; *p* = 0.083
Edinburgh handedness inventory (Swedish version), non‐right handedness, *n* (%)	11(20)	12 (21)	−0.04; *p* = 0.890
Intracranial volume, l, M (SD)	1.60 (0.12)	1.67 (0.15)	**−0.53; *p* < 0.001**
Education, highest level, *n* (%)			
Primary school ≤9 years	6 (11)	2 (4)	−0.36; *p* = 0.164
Secondary school 1–3 years	26 (47)	23 (40)	
Postsecondary education	23 (42)	32 (56)	
Employed, *n* (%)	32 (58)	50 (88)	**−0.90; *p* < 0.001**
Pedophilic sexual attraction			
Age of discovery of pedophilic sexual attraction, yrs, range, M (SD)^d^	6–39, 18 (7)	NA	–
Attraction primarily to boys, *n* (%)	8 (15)	NA	–
Attraction primarily to girls, *n* (%)	43 (78)	NA	–
Attraction to boys and girls, *n* (%)	4 (7)	NA	–
Attraction exclusively to prepubescent children, *n* (%)	12 (22)	NA	–
Self‐reported convictions, *n* (%)			
Any sexual offence^i^	12 (22)	1 (2)	**1.52; *p* < 0.001**
Contact sexual offence (child)^e^	5 (9)	0 (0)	**0.96; *p* = 0.026**
Non‐contact sexual offence (child or adult) or CSEM offence^e^	8 (15)	0 (0)	**1.25; *p* = 0.003**
Any non‐sexual offence	8 (15)	13 (23)	−0.30; *p* = 0.263
Adult‐related sexual orientation, *n* (%)^f^			
Heterosexual	30 (71)	45 (83)	0.17; *p* = 0.328
Homosexual	4 (10)	4 (7)	
Bisexual	8 (19)	8 (15)	
Victim of sexual abuse before age 15, *n* (%)	6 (11)	6 (11)	0.02; *p* = 0.948
Mini International Neuropsychiatric Interview 6.0.0, *n* (%)^e^			
Major Depressive Episode (current)	14 (26)	0 (0)	**1.64; *p* < 0.001**
Major Depressive Episode (current and previous)	6 (11)	0 (0)	**1.07; *p* = 0.012**
Major depressive episode (previous)	1 (2)	3 (5)	−0.61; *p* = 0.618
Dysthymia (current)	10 (18)	0 (0)	**1.40; *p* < 0.001**
Manic episode (current)	0 (0)	0 (0)	**–**
Manic episode (previous)	1 (2)	0 (0)	0.03; *p* = 0.491
Hypomanic episode (current)	1 (2)	0 (0)	0.03; *p* = 0.491
Hypomanic episode (previous)	2 (4)	0 (0)	0.42; *p* = 0.239
Panic disorder (current)	4 (7)	0 (0)	0.83; *p* = 0.055
Panic disorder (previous)	3 (6)	1 (2)	0.65; *p* = 0.359
Social anxiety disorder (current)	16 (29)	0 (0)	**1.74; *p* < 0.001**
Generalized anxiety disorder (current)	8 (15)	2 (4)	0.85; *p* = 0.051
Obsessive‐compulsive disorder (current)	10 (18)	0 (0)	**1.40; *p* < 0.001**
Post‐traumatic stress disorder (current)	1 (2)	0 (0)	0.03; *p* = 0.491
Psychotic syndrome (current)	1 (2)	0 (0)	0.03; *p* = 0.491
Any psychotic symptoms (current)	2 (4)	0 (0)	0.42; *p* = 0.239
Anorexia nervosa (current)	0 (0)	0 (0)	–
Bulimia nervosa (current)	0 (0)	1 (2)	0.01; *p* = 1.00
Antisocial personality disorder (lifetime)	9 (16)	1 (2)	**1.32; *p* = 0.008**
Psychoactive medication, *n* (%)			
Antidepressant	15 (27)	3 (5)	**1.05; *p* = 0.002**
Other^g^	12 (22)	3(5)	**0.90; *p* = 0.009**
Ritvo Autism and Asperger Diagnostic Scale – screening tool^h^			
Total sum (range 0–42), Mdn (IQR)	18 (20)	4 (5)	**0.99; *p* < 0.001**
Mentalizing subscale score (range 0–21), Mdn (IQR)	10 (12)	2 (3)	**0.98; *p* < 0.001**
Sensory reactivity subscale score (range 0–9), Mdn (IQR)	3 (4)	0 (3)	**0.77; *p* < 0.001**
Social anxiety subscale score (range 0–12), Mdn (IQR)	4 (8)	1 (3)	**0.88; *p* < 0.001**
Total score >13, *n* (%)	32 (60)	5 (9)	**1.57; *p* < 0.001**
Total score >22, *n* (%)	17 (32)	2 (4)	**1.41; *p* < 0.001**
Adult ADHD Self‐Report Scale – Screen (range 1–6), total score >3, *n* (%)^h^	20 (38)	9 (16)	**0.65; *p* = 0.009**
Alcohol Use Disorders Identification Test (range 0–40), total score >7, *n* (%)^h^	6 (11)	9 (16)	−0.21; *p* = 0.495
Drug Use Disorders Identification Test (range 0–44), total score >5, *n* (%)^e^, ^h^	4 (8)	2 (4)	0.45; *p* = 0.426
Hypersexual Behavior Inventory (range 19–95), total score >52, *n* (%)^h^	33 (62)	1 (2)	**2.50; *p* < 0.001**
Wechsler Adult Intelligence Scale–IV^b^			
Full‐scale IQ, Mdn (IQR)	101 (23)	114 (15)	**−0.82; *p* < 0.001**
Verbal comprehension index, Mdn (IQR)	104 (23)	118 (14)	**−1.02; *p* < 0.001**
Perceptual reasoning index, Mdn (IQR)	110 (18)	113 (20)	−0.29; *p* = 0.127

Note

For reasons of completeness, we exchanged a frequency of 0 to 1 in effect size calculations to obtain Cohen's *d* estimates.

Abbreviations: CSEM, child sexual exploitation material; NA, not applicable.

^a^ Bolded figures are significant at *p* < 0.05 (two‐tailed).

^b^ Missing data from 1 HC.

^c^ Missing data from 1 PD participant.

^d^ Four PD participants reporting they “had always known” about their pedophilic sexual attraction were excluded from this comparison.

^e^ Expected counts were <5 in one or more cells and *p*‐values calculated with Fisher's Exact Test.

^f^ PD participants exclusively attracted to children were excluded (*n* = 12), missing data from 1 PD and 3 HC participants.

^g^ Other psychoactive medications included stimulants, mood stabilizers, and sleep medication, e.g., antihistamines.

^h^ Missing data from 2 PD participants.

^i^ One PD patient reported both contact and non‐contact sexual offences.

Pedophilic disorder and HC individuals differed numerically in BMI and education level. However, these differences were not statistically significant. PD patients had moderately and significantly smaller intracranial volume than HC subjects and were more often unemployed. There were marginal but non‐significant differences in adult‐oriented sexual orientation and sexual abuse victimization before age 15. Compared with HC, substantially larger proportions of PD men self‐reported sexual offending and had additional psychiatric disorders. Specifically, more individuals with PD had major depressive episodes, dysthymia, social anxiety disorder, obsessive‐compulsive disorder (OCD), and antisocial personality disorder. More individuals with PD were currently taking an antidepressant or another psychoactive medication, respectively. Significantly more PD than HC participants reported clinically relevant levels of hypersexuality, ADHD, and ASD symptoms; effect sizes were moderate to large. Albeit PD patients were on average still within the population average range (93–107),^22^ PD subjects had significantly lower IQ than HC participants, effects were large and driven mainly by differences in verbal comprehension. We found that 2D:4D ratios were slightly but non‐significantly larger in PD than in HC men. We tested the effects of demographic and clinical variables on main neuroimaging outcomes in sensitivity tests.

### 3.2 Case‐control differences in structural brain imaging measures (vertex‐wise main analysis)

**FIGURE 1:**
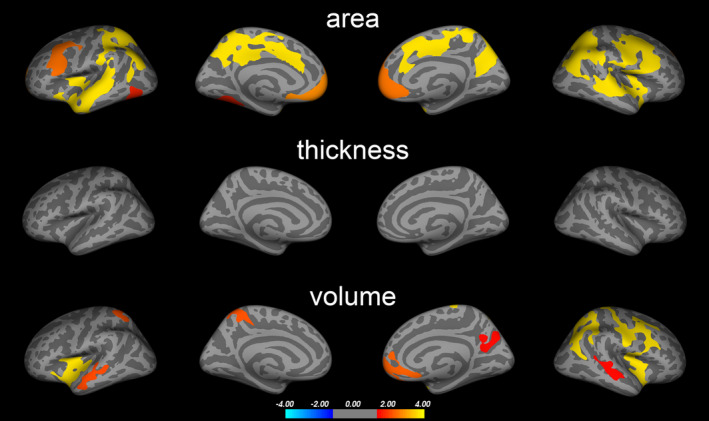
Brain abnormalities in PD (main analysis). Colored clusters represent areas in which significant differences in cortical surface area, thickness, and volume were found between PD and HC subjects. Data underwent Monte Carlo cluster‐wise corrections for multiple comparisons (see Figure S1 for uncorrected results). Statistical significance is displayed on a log(p)‐scale where positive values (warm colors) represent the PD < HC contrast. No negative values (cold colors) reflecting PD > HC patterns were observed. Cluster‐wise statistics are given in Table S1

Figure 1 depicts results of vertex‐wise cortical structure analyses. PD showed smaller cortical surface area in clusters comprising (i) bilateral ventromedial prefrontal cortex (vmPFC, also including medial superior frontal, medial orbitofrontal, and rostral anterior cingulate cortex), (ii) caudal and posterior cingulate cortex, precuneus and paracentral cortex, supramarginal and superior/inferior parietal cortex, (iii) left fusiform/inferior temporal and lingual cortex (iv) middle and superior temporal cortex, (v) insula, lateral prefrontal cortex (pars opercularis/triangularis, caudal/rostral middle frontal), extending into pre‐ and postcentral cortex. Yet not as pronounced, PD subjects also showed lower cortical volume in regions largely in agreement with those where surface area abnormalities were found. We observed no group differences in cortical thickness. A more detailed description, corresponding cluster statistics, and uncorrected results are available in the Supporting Information (Figure S1, Tables S1 and S2).

### 3.3 Case‐control differences in subcortical and WM volume (secondary analyses)

Compared with HC, PD subjects demonstrated lower (bilateral) hippocampal and right accumbens volumes (Table S3). PD showed lower WM volumes in areas that largely align with brain regions in which cortical WM‐surface area abnormalities were found (Table S4). No PD > HC patterns were present.

### 3.4 Sensitivity tests

Our results generally remained robust when correcting for demographic and clinical variables, with a few exceptions; for example, when correcting for intracranial volume (ICV) or IQ, several group comparisons of WM and subcortical volumes were no longer statistically significant. We want to emphasize that controlling for ICV and IQ may indirectly control for the PD phenotype and disguise effects of interest, which may explain the observed decrease in significance in some brain areas.^27^ Hence, results obtained when correcting for these factors should be interpreted with caution. When correcting for sexual offending, group differences in WM volume underlying right caudal anterior cingulate, right paracentral, and left posterior cingulate cortex were no longer statistically significant. Results of sensitivity tests are presented in more detail in the Supporting Information (Table S5 and S6).

### 3.5 Correlations with 2D:4D

**FIGURE 2:**
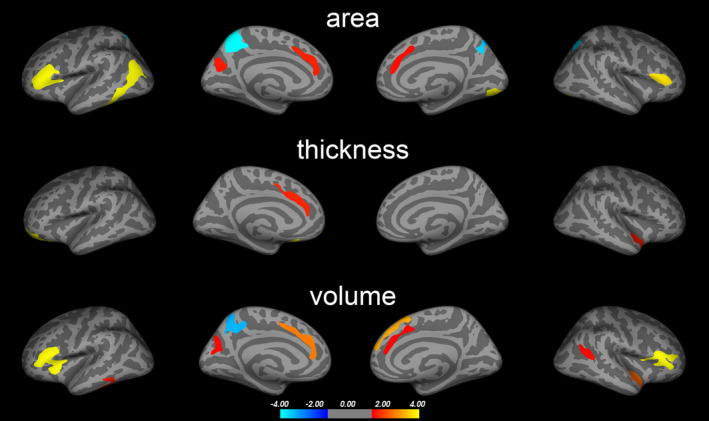
2D:4D correlations. Correlations between 2D:4D ratios and cortical surface area, thickness, and volume in PD. Colored areas represent brain regions in which significant correlations were observed after multiple comparison correction (see Figure S3 for uncorrected results). Statistical significance is displayed on a ‐log(p) scale. Cold colors represent negative and warm colors positive correlations, respectively

**FIGURE 3:**
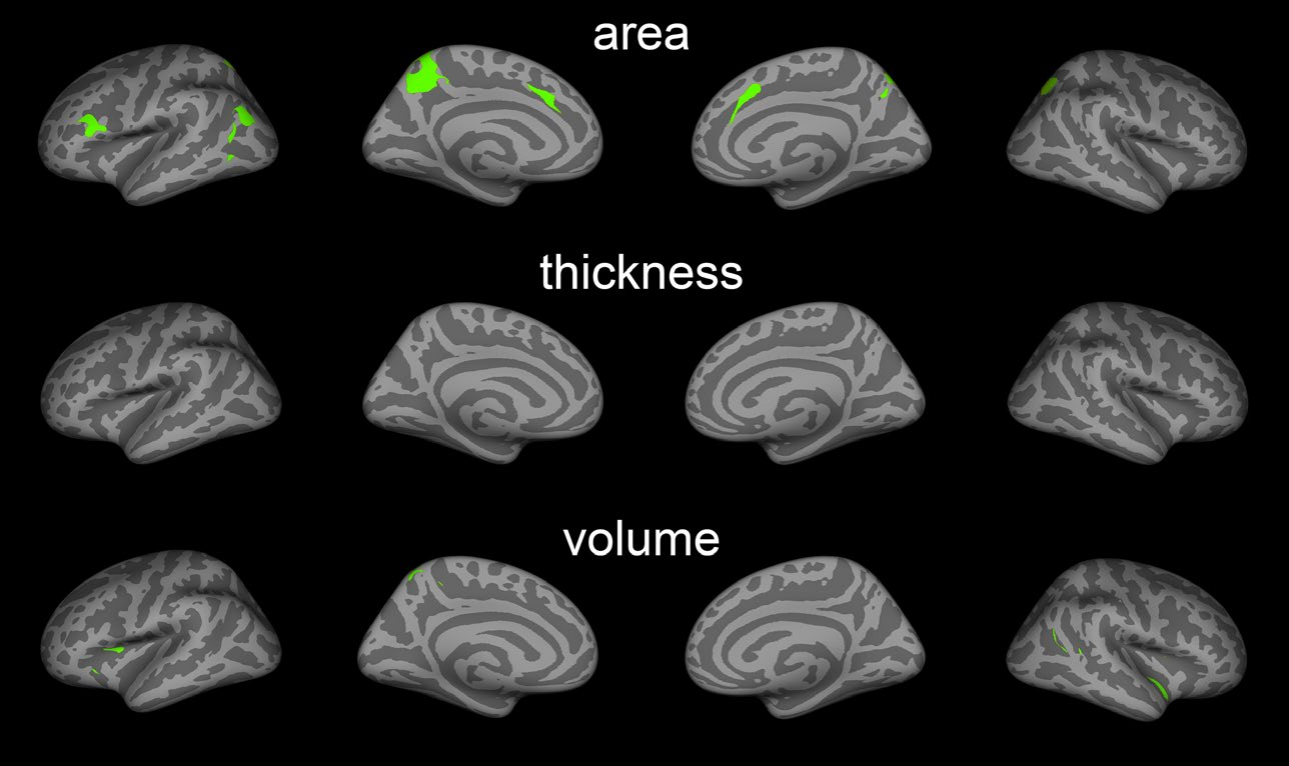
2D:4D correlations and PD‐abnormality conjunction maps. Green areas indicate the regional overlap between areas where 2D:4D correlations (Figure 2) and PD‐related abnormalities were found (Figure 1)

We found both significant positive and negative correlations between 2D:4D and cortical surface area, thickness, and volume in PD patients (Figure 2). These areas largely overlapped with regions in which PD‐related abnormalities were found (Figure 3). The regions included the inferior frontal cortex (pars triangularis, pars opercularis; positive), precuneus (negative), medial superior frontal and caudal ACC (positive), superior parietal (negative), inferior parietal (positive), middle and inferior temporal (positive), and the insula cortex (positive). In line with this, we also found suggestive correlations between 2D:4D and WM volume underlying the right caudal ACC (positive) and bilateral superior parietal volumes (negative) (Table S8).

### 3.6 Multivariate classification and pattern matching

**FIGURE 4:**
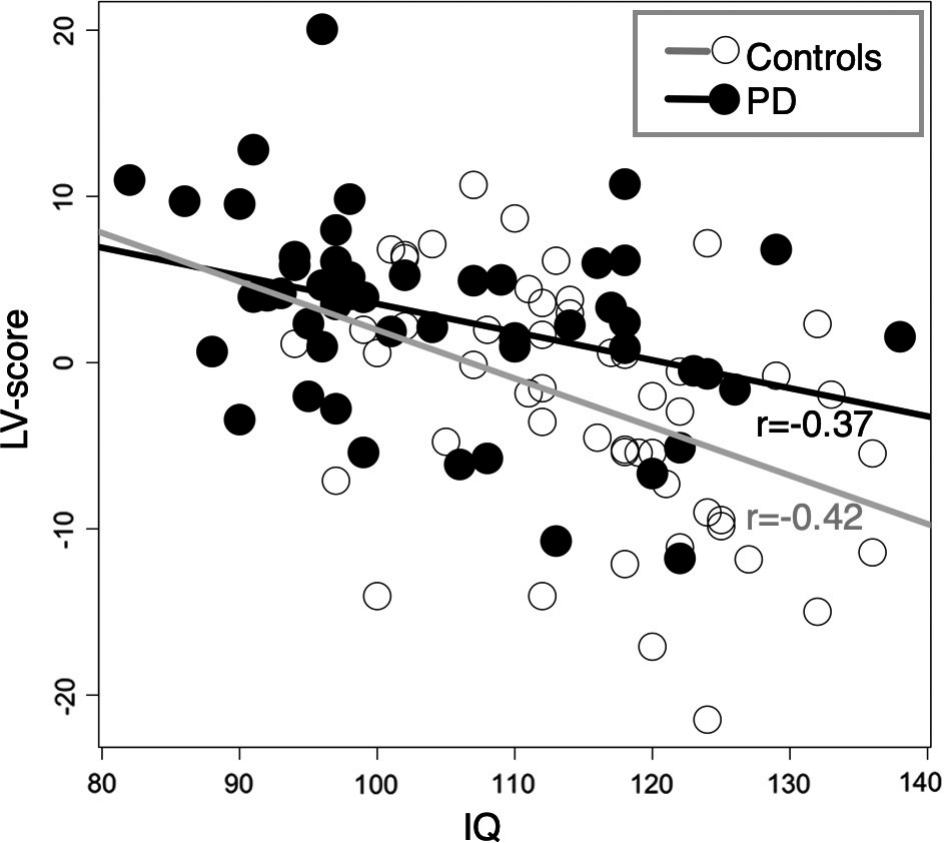
Correlation between global morphometric PD abnormality expression score (LV‐score) and IQ. Relationships in this plot are quantified with Pearson correlation coefficients: *r* = −0.37 (*p* = 0.005) and *r* = −0.42 (*p* = 0.001) for PD and control groups, respectively

IQ exhibited a consistent negative association with the global morphometric PD abnormality expression score, which was characterized mainly by reductions in parietal, temporal, and prefrontal volumes (Figure S6). The relationship between the global morphometric PD abnormality expression score and IQ was present in the full sample and within PD and HC groups, respectively (Table S7 and Figure 4), indicating that associations are not driven by group differences.

## 4 DISCUSSION

In this single‐center study of pedophilic disorder (PD), we present a PD profile from detailed characterization of clinical symptomatology, cognitive functioning, and brain abnormalities. Brain measures included cortical thickness, surface area, and volume, as well as subcortical and regional white matter (WM) volumes. Additionally, we investigated associations between brain structure and IQ as well as prenatal androgen exposure (2D:4D).

### 4.1 Indications for neurodevelopmental contributions to PD

We found that PD was associated with smaller cortical surface area in several brain regions. Overall, these findings remained when adjusting for IQ variability. In contrast, no group differences in cortical thickness were observed. Our analyses revealed that cortical surface abnormalities were more pronounced in the vicinity of WM (Figure S4). Consistent with this, we observed smaller regional WM volumes in patients with PD compared to HC participants in regions where surface abnormalities were found. This agrees with a previous study that reported prefrontal, microstructural WM matter alterations in PD using diffusion tensor imaging.^28^ The exclusive alteration of surface area, but not cortical thickness, in PD could be related to the fact that these two imaging phenotypes are influenced by distinct developmental mechanisms.^29^, ^30^ For example, genetic influences on cortical thickness and surface area appear to differ.^29^, ^30^ Thus, our findings suggest that mechanisms affecting surface area may play a role in PD. Although the exact mechanisms behind determination of cortical thickness and surface area remain largely unknown, they are believed to exert effects during the pre‐ and perinatal periods of neurodevelopment.^29^, ^30^, ^31^

Pedophilic disorder has been suggested to be associated with aberrant neurodevelopment.^2^, ^3^, ^4^ Consistent with that notion, we found that the 2D:4D ratio, a marker of prenatal androgen exposure, correlated with both cortical surface area and WM volume in those brain regions where PD differed from HC participants. Thus, our findings support prenatal androgen exposure as a possible determinant of the PD neural signature. Androgen effects have been linked to myelination,^32^ affecting WM volume and in turn cortical surface area, but not thickness.^33^ Although this is a plausible neurodevelopmental explanation to our findings, the exact mechanisms that contribute to the brain abnormalities found and the origin of the observed correlations are likely complex and cannot be derived from this study. Further research is needed to investigate this emerging hypothesis.

Moreover, we found lower IQ in PD compared with HC men, primarily driven by differences in verbal comprehension, and the expressed PD‐related brain abnormality pattern correlated negatively with total IQ. This is in agreement with a previous study reporting that IQ‐mediated cortical differences between controls and individuals with pedophilic interest.^13^ Since IQ is highly influenced by neurodevelopmental factors, this may suggest that the overall PD‐related structural abnormality pattern could arise from neurodevelopmental mechanisms. This possibility, however, and the mediating mechanisms behind the link between IQ and PD‐related brain abnormalities remain unsettled.

Moreover, PD patients expressed clinical symptoms of the neurodevelopmental disorders ADHD and ASD.^1^ Previous studies have reported higher prevalence of ADHD among individuals with PD. Here, we demonstrate that this also applies to ASD symptoms. Interestingly, developmental trajectories of cortical surface area, but not thickness, have been shown to deviate from normal in autism.^34^ This aligns with the present findings of different cortical surface area but not thickness. Although autistic traits may be highly relevant for the clinical profile of PD, we controlled for it in sensitivity analyses, indicating that observed structural brain abnormalities were specifically related to PD and not to ASD symptoms per se.

### 4.2 Brain regions affected in PD

Although we did not replicate previously reported amygdala alterations in PD,^6^ our cortical surface area findings largely agreed with those of a recent multi‐center study,^13^ not only with respect to outcome measure but also to anatomical location. However, the brain abnormalities observed in the previous study were reportedly related to offender status rather than pedophilic interest. This discrepancy could be explained by differences in study design and analytical strategies, but also by limited statistical power and potential confounding both due to the merging of small samples investigated with numerous different MRI scanners in previous studies. We were able to use the same MRI scanner and controlled for several potential confounds, including sexual offending. Our sensitivity analyses indicated that the abnormalities reported here were indeed related to PD, and not to sexual offender status or other clinical and demographic variables.

Specifically, we found PD‐related abnormalities in the ventromedial prefrontal cortex (vmPFC), posterior cingulate, precuneus, and inferior parietal lobule, including the angular gyrus and supramarginal cortex. These brain structures are important hub regions of the brain's default mode network (DMN).^35^, ^36^, ^37^ The DMN also contains the lateral middle temporal cortex, temporal pole, temporoparietal junction (TPJ), and hippocampus^35^, ^36^, ^37^; regions also altered in PD participants. Hence, the structural abnormality pattern observed in PD occurs primarily in DMN regions. This network is involved in internal and self‐related processes, but also in social cognition including thinking about others, their emotions and thoughts.^35^, ^36^, ^37^ The DMN is also recruited when imagining future consequences of one's own actions and moral reasoning.^35^, ^36^, ^37^ Intriguingly, the DMN is impaired in several psychiatric conditions, including ADHD and ASD, and putatively involved in the clinical expression of these disorders.^37^ Notably, the DMN also interacts with other brain regions and networks with similar functions. For example, PD subjects showed abnormalities in the lateral prefrontal and anterior insula cortex, key regions for cognitive control, decision‐making, social cognition, and integration of self‐related and emotional processes.^38^, ^39^ Behavioral aspects reportedly associated with PD in forensic samples include impulsivity and risk taking as well as impaired social skills, empathy, moral reasoning, and self‐reflection.^4^, ^40^ Hence, we speculate that the brain abnormalities observed here may relate to increased PD‐related behaviors. However, this cannot be concluded from this study, and may only apply to a sub‐group of patients. Notably, not all PD patients showed pronounced ASD symptoms. Further, it remains unknown how observed brain abnormalities relate to the development of the core symptom of PD; the sexual attraction to prepubescent children. Within the PD group, we observed brain differences related to pedophilic sexual orientation (sexual attraction toward girls and/or boys), which do not appear to align with regions commonly associated with adult‐related sexual orientation^41^ (Figure S2). The present findings may constitute initial steps toward understanding the complex interplay of neurobiological factors underlying sexual attraction patterns in PD.

In conclusion, in the largest single‐center study to date, we investigated self‐referred, help‐seeking patients with DSM‐5 PD in a non‐correctional, clinical setting. Our results suggest that PD co‐occurs with substantial psychiatric morbidity including depressive and anxiety disorders, clinical symptoms of ADHD and ASD, and somewhat lower IQ, albeit still in the normal range. We report that PD is related to cortical surface area alterations and underlying white matter abnormalities, primarily in brain regions belonging to the default mode network. The results were not confounded by demographic or clinical variables, and importantly, unrelated to self‐reported sexual offence history. The neuroanatomical findings, their association with IQ and 2D:4D, and the symptom profile suggest that PD may have neurodevelopmental underpinnings. The results could inform the development of preventive and supportive interventions for PD patients.

### 4.3 Strengths and limitations

This study is of substantial clinical relevance through the comprehensive characterization of a representative clinical sample of self‐referred and help‐seeking men with PD. Potential limitations are that structural neuroimaging cannot assess the functional involvement of brain regions in the expression of PD. Functional MRI studies are necessary to quantify the neural correlates of cognitive and behavioral aspects of PD. Second, the cross‐sectional study design does not allow for inference regarding causality. Third, it is unknown whether PD patients with psychiatric comorbidity, including symptoms of ASD, are more prone to seek help. However, the investigated sample is representative for self‐referred individuals with PD in a clinical, non‐correctional setting. Finally, although a history of sexual offending behavior did not explain our findings, prospective studies following PD patients over longer periods of time might help identify clinical, cognitive, and neurobiological variables that relate to the risk of child sexual abuse.

## CONFLICT OF INTEREST

None.

## AUTHORS’ CONTRIBUTIONS

CR and BL contributed with study design and conceptualization. CA conceived analyses, performed most brain imaging analyses, and wrote the first draft of the manuscript. RA planned and performed analyses of clinical and behavioral measures together with NL. CM performed analyses on white matter and subcortical measures and AL conducted classification analyses. CA, RA, NL, CM, and AL created tables and figures. All authors contributed with interpretation of data, critically revised the manuscript for intellectual content, approved the final version of the manuscript for publication, and agree to be accountable for all aspects of the work.

### Peer Review

The peer review history for this article is available at https://publons.com/publon/10.1111/acps.13273.

## Supporting information

Supplementary Material

## Data Availability

Data access and codes can be obtained on reasonable request.
