# Supplementary material for: Brain structure and clinical profile point to neurodevelopmental factors involved in pedophilic disorder
Source: Acta Psychiatr Scand. 2021 Jan 22;143(4):363–74. doi: 10.1111/acps.13273 (PMC7986195; doi:10.1111/acps.13273)
Supplement: Supplementary file 1 — Supplementary Material [file ACPS-143-363-s001.docx]

Supplementary Material for

**Brain structure and clinical profile point to neurodevelopmental factors involved in pedophilic disorder.**

Christoph Abé^1^, Roberth Adebahr^2^, Benny Liberg^1^, Christian Mannfolk^3^, Alexander Lebedev^1^, Jonna Eriksson^4^, Niklas Långström^5^ and Christoffer Rahm^3^

Correspondence to: Christoph Abé, christoph.abe@ki.se

**Supplementary Materials and Methods**

*Participants recruitment and screening*

We performed this study within the framework of the *Pedophilia at Risk - Investigations of Treatment and Biomarkers* (PRIOTAB) research project, evaluating effects of the gonadotropin-releasing hormone (GnRH) antagonist degarelix on risk factors for committing child sexual abuse in a randomized placebo-controlled trial (PRIOTAB-RCT) [^1^](#_ENREF_1). The study was conducted at ANOVA, a center for sexual medicine, andrology, and transgender medicine at Karolinska University Hospital, Stockholm, Sweden. Subjects with PD were recruited consecutively from March 2016 to April 2019 through PrevenTell, a Swedish national helpline for “unwanted sexual desires” administered by specialized sexual medicine professionals at ANOVA. Subjects expressing concern about their sexuality underwent a semi-structured telephone-based screening for paraphilic interests and behaviors, compulsive sexual and criminal behavior. Potential PRIOTAB participants (i.e., help-seeking individuals reporting sexual interest in prepubescent children) were re-contacted by phone by a board-certified adult psychiatrist who provided further study information and assessed patient eligibility. PD was confirmed using a structured interview based on DSM-5 criteria. Males with PD between 18 and 66 years of age without current severe psychotic symptoms, high suicide risk, or severe substance abuse were eligible. Age-matched healthy male controls (HC), screen-negative for PD, were recruited through Karolinska Trial Alliance (https://karolinskatrialalliance.se) using the same exclusion criteria. Eligible subjects were invited to the ANOVA outpatient clinic for written informed consent, and a comprehensive baseline work-up including semi-structured psychiatric diagnostic interviews, self-report questionnaires, anthropometric measurements, neuropsychological testing, blood sampling, and brain MRI. PD patients were encouraged to take part in treatment as usual after completing the RCT. Participants were not previously known to the psychiatrist interviewer. More details on screening and recruitment procedures can be found elsewhere [^1^](#_ENREF_1). Fifty-five adult men with PD and 57 healthy controls (HC) completed baseline clinical assessments of psychiatric comorbidity, attention deficit hyperactivity disorder (ADHD) and autism spectrum disorder (ASD) symptom self-reports, anthropometric characteristics, and general cognitive functioning (IQ).

*Structural MRI image acquisition and processing*

We performed brain magnetic resonance imaging (MRI) scans at the Karolinska University Hospital, Huddinge, Sweden, using a 3T medical scanner (Siemens Prisma) equipped with a 64-channel head coil. T1-weighted anatomical images were acquired with a magnetization prepared rapid acquisition gradient echo sequence (MP-RAGE; 176 slices; TR = 1900 ms; TE = 2.52 ms; voxel size = 1×1×1 mm^3^). The MRI scan was performed before the PRIOTAB RCT started. A board certified neuroradiologist examined MRI images to rule out signs of neuropathology. We obtained measures of cortical volume, thickness and surface for each participant from structural T1-weighted images using the semi-automated cortical surface reconstruction and parcellation methods provided by FreeSurfer 6.0 [^2-6^](#_ENREF_2). The procedure includes intensity normalization, removal of non-brain tissue, segmentation of cortical grey, subcortical white, deep grey matter volumetric structures, and triangular tessellation of the grey/white matter interface (WM surface) and white matter/cerebrospinal fluid boundary (pial-surface). Before data extraction, segmentations and reconstructions of the surfaces were visually inspected and, if necessary, corrected manually using FreeSurfer editing tools. For surface-based vertex-wise analyses, individual reconstructed surfaces were smoothed (10 mm), transformed, and resampled onto a common standard space (fsaverage) using the -qcache command. FreeSurfer also provided segmentation of subcortical volumes (amygdala, thalamus, putamen, nucleus accumbens, pallidum, caudate, and hippocampus), and cortical parcellation of 34 regions of interest (ROIs) per hemisphere (average regional cortical thickness, surface area, and volume as defined by the Desikan atlas [^7^](#_ENREF_7)), and segmentation of white matter (WM) volumes of regions underlying these cortical parcellations. These measures were extracted for secondary analyses, including multi-variate morphometric covariance pattern extraction for PD vs. HC classifications (described below).

*Statistical analyses*

*Case-control differences in structural brain imaging measures.* To quantify group differences in brain imaging measures on vertex level, statistical maps were computed using a general linear model approach at each vertex point of the cortical surface using analysis tools provided by FreeSurfer (*mri_glm_fit*). In the main analysis, we tested for the effect of group (PD vs HC; independent variable of interest) on cortical thickness and WM surface are area (dependent variables), while correcting for age (regressor of no interest). Cortical volume comparisons were performed for completeness. Correction for multiple comparisons was done using a Monte Carlo cluster-wise simulation approach (threshold of p=0.05) considering two spaces (hemispheres) [^8^](#_ENREF_8). Results were loaded into QDEC for visualization.

Secondary tests on subcortical volumes and regional WM segmentations were conducted in SPSS using multiple univariate analyses of covariance (ANCOVAs). Brain imaging phenotypes were set as dependent variables, group was entered as fixed factor, and age as covariate. Multiplicity of ANOVA tests was considered using the false discovery rate (FDR). Mean differences between HC and PD subjects were quantified with Cohen’s *d*.

*Sensitivity tests (testing for potential confounds on brain imaging findings).* For sensitivity analyses testing for potential confounding factors, we extracted cortical thickness, surface area, and volume measures averaged over significant clusters obtained in the main analysis using *mri_segstats.* First, we compared these measures between groups to validate our main findings. This was done using the same statistical model as described for subcortical and WM volume analyses but using the extracted brain measures as dependent variable (ANCOVA, SPSS, Supplementary Table 2). We then tested for potential confounding effects by demographic or clinical variables (e.g. comorbidities, medication use) on group differences by entering them as additional covariates (one at a time) in the statistical model. In additional sensitivity tests, when fewer than ten participants had a specific comorbidity/medication, we repeated confounder analyses one at a time after having excluded those individuals. Specifically, we performed follow-up analyses separately controlling for body mass index (BMI), handedness, IQ, sexual abuse victimization before age 15, sexual offending (any sexual offence), psychiatric comorbidities, AUDIT score, DUDIT score, hypersexual behavior, pronounced ASD and ADHD symptoms, adult-related sexual orientation, antidepressant use, and other psycho-active medication use. We performed additional analyses after excluding cases with panic disorder, psychotic symptoms, hypomania, mania, dysthymia, PTSD, bulimia, PD patients reported contact sexual offence, PD patients who reported non-contact sexual offence or CSEM offence, or PD patients with pedophilic sexual attraction towards boys only or both boys and girls, respectively. Among PD patients we also compared exclusive PD (no sexual attraction to adults) with non-exclusive PD (sexual attraction to children and adults), those with and without pronounced ASD symptoms, and PD patients with pedophilic sexual orientation (PSO) towards prepubescent girls (n=39, g-PSO) with those attracted to boys or both boys and girls (n=11, b-PSO). Corresponding analyses were performed for subcortical and WM volume measures.

*Vertex-wise correlations with 2D:4D (interpretational purpose).* To investigate the potential role of prenatal androgens on cortical structure in PD, we tested for regional associations between 2D:4D and cortical measures by computing statistical maps using a general linear model approach for the effects of 2D:4D (covariate of interest) on cortical thickness, area, and volume (dependent variable) at each vertex point of the cortical surface, while correcting for age. Although this was a secondary analysis, we performed correction for multiple comparisons using a Monte Carlo cluster-wise simulation approach as described above. To visualize regional overlaps between 2D:4D correlation maps and PD-related cortical alterations identified in the main analysis (case-control comparisons), we computed conjunction maps between corresponding Monte Carlo corrected maps using *mri_concat*. Finally, we also computed partial correlations between 2D:4D and subcortical and WM volumes, while correcting for age.

*WM-surface and pial-surface area comparisons (interpretational purpose).* Given our volumetric white matter and cortical WM surface area findings, we tested if cortical surface area alterations are more or less pronounced at the white matter/cerebrospinal fluid boundary (pial-surface) compared with grey/white matter interface (WM surface; main analysis). Therefore, we performed case-control comparisons on vertex level using pial-surface area as the dependent variable (Supplementary Figure 4).

*Multivariate pattern and classification analysis.* To test whether global morphometric brain patterns expressed in PD are linked to clinical and behavioral characteristics, we used partial least squares (PLS) classification [^9^](#_ENREF_9). PLS is a projective data-driven method of dimensionality reduction and multivariate feature extraction, used widely in various practical applications including clinical diagnostic and imaging classification problems [^10^](#_ENREF_10). As structural variables, we used all outputs of FreeSurfer parcellated regional cortical surface area, WM volumes underlying each of the cortical regions (according to the Desikan atlas), and subcortical volumes. The PLS method projects a high‐dimensional feature space onto a small set of latent variables (LVs), each characterizing a distinct multivariate covariance pattern. For each subject, extracted (LV) scores characterize the magnitude of expression of the identified PD-related abnormality pattern (here referred to as global morphometric PD abnormality expression score). We also tested the effects of clinical and neuropsychological variables on morphometric PD scores in a multivariate regression model (pattern matching), adjusting for age. We tested associations with 2D:4D, total IQ, and the six variables showing effects on our outcome measures in the main analysis; age, group status (PD vs HC), sexual offender status, social anxiety disorder, antidepressant use, and hypersexual behavior. Association analyses were repeated when correcting for PD vs HC status, and within cases and controls separately, to rule out a potential confound by group status. Classification analyses were performed in R.

**Supplementary Results**

*Sensitivity tests*

For all MRI-derived measures, results generally remained robust when correcting for demographic and clinical variables and after excluding individuals with a specific comorbidity or medication use. There were few exceptions:

When correcting for intracranial volume (ICV), differences in left area clusters 3 (medial prefrontal, p=0.072) and 5 (fusiform, p=0.063) were no longer statistically significant (Supplementary Table 5). When adjusting for IQ, the same applied to left area clusters 3 (medial prefrontal, p=0.070) and 5 (fusiform, p=0.077), and right area cluster 4 (medial prefrontal, p=0.095). Results of sensitivity tests of cortical measures are presented in more detail in Supplementary Table 5. Controlling for ICV and IQ affected most group differences in regional WM volume (Supplementary Table 6). Notably, we argue that ICV and IQ are related to the PD phenotype. Thus, controlling for ICV and IQ may indirectly control for PD and disguise effects of interest, which may explain the observed decrease in significance in some brain areas [^11^](#_ENREF_11).

When correcting for sexual offending (any sexual offence), group differences in WM volume underlying right caudal anterior cingulate, right paracentral, and left posterior cingulate cortex are no longer statistically significant (Supplementary Table 6). However, results held when excluding PD patients reporting contact sexual offences (n=5) and when excluding PD reporting non-contact sexual offence or CSEM offence (n=8).

No group differences in hippocampal volumes were observed when correcting for hypersexual behavior (Supplementary Table 6).

We found no differences between subjects with exclusive and non-exclusive PD, nor between those with and without pronounced ASD symptoms. In PD patients, we found a significant effect of pedophilic sexual orientation (PSO) on volume in the right middle/superior temporal cortex (F(47,1)=9.26, p=0.004) and WM volume in the right caudal ACC (F(47,1)=4.84, p=0.033). This was further explored in follow-up tests to exclude potential confounding by PSO (see below).

*Pial surface area and WM surface area comparisons (interpretational purpose)*

PD-related pial surface abnormalities were less pronounced than abnormalities found in WM-surface area (supplemental Supplementary Figure 4).

*Multivariate classification and pattern matching*

Plotting root mean squares of the prediction error as a function of LV number, we identified one LV (explaining 40% of the total variance in the feature space) as sufficient to provide good classification performance, which resulted in a sensitivity of 0.72, and specificity of 0.65 (AUC = 0.71) (Supplementary Figure 5).

**Note**: considering the accuracy of the classification analysis, the potential multifactorial mechanisms at play, and the existence of PD subtypes, our findings do not allow the prediction of PD based on neuroimaging measures.


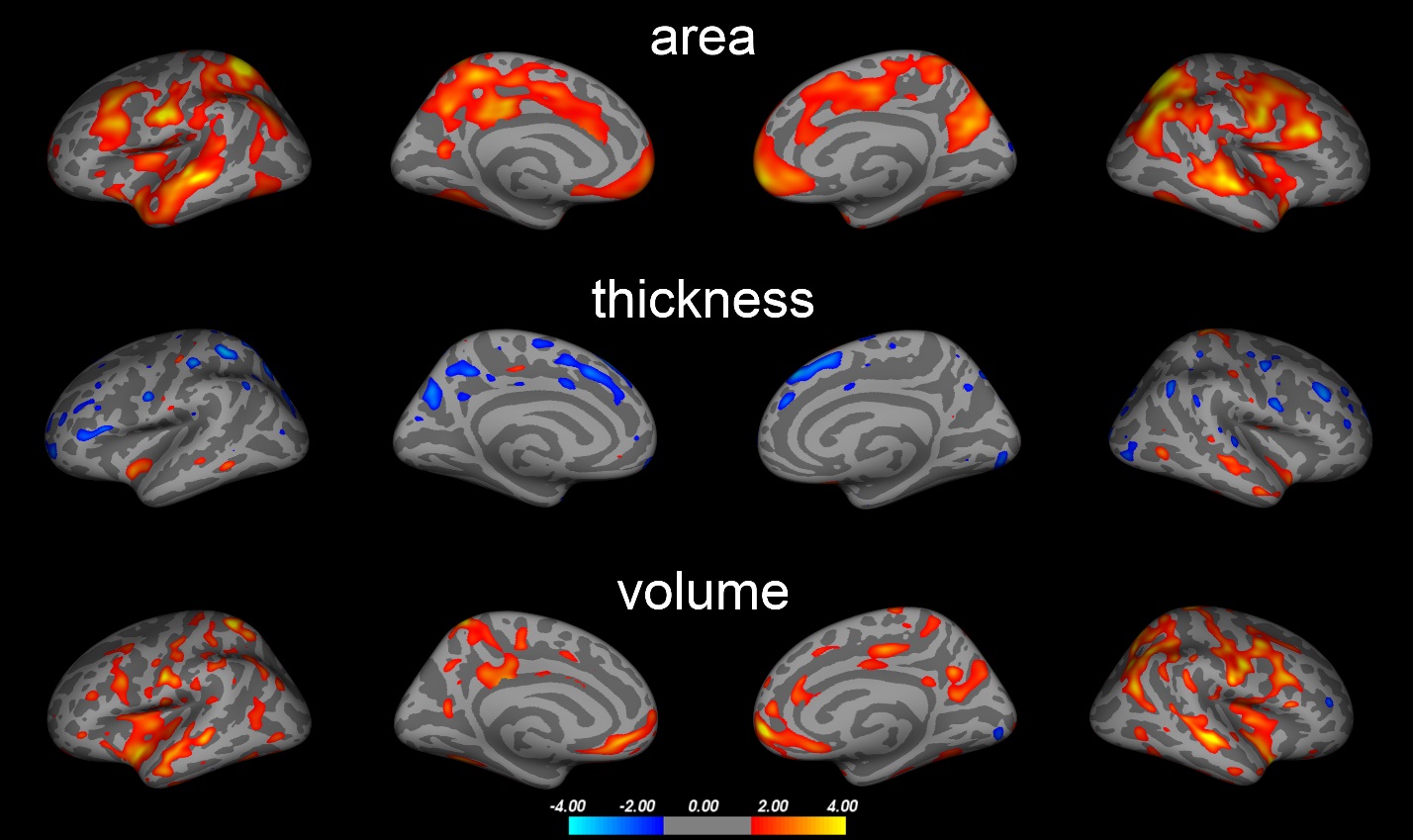


**Supplementary Figure 1:** **Uncorrected main analysis.** Clusters represent areas in which significant differences in cortical surface area, thickness, and volume were found between PD and HC (uncorrected threshold at p<0.05). Statistical significance is displayed on a log(p)-scale were positive values (warm colours) represent the PD < PD and negative values the HC< PD contrast.

**Supplementary Table 1: Cluster summary statistics of vertex-wise main analysis.**

| **Hemisphere** | **Measure** | **Cluster** | **Size (mm^2^)** | **CWP** | **Max vertex** | **MNI X** | **MNI Y** | **MNI Z** | **Desikan label (max. vertex)** | **Cluster includes** |
| --- | --- | --- | --- | --- | --- | --- | --- | --- | --- | --- |
| left | area | 1 | 10675.30 | 0.0002 | 5.308 | -29.9 | -56.0 | 61.9 | superiorparietal | inferior parietal supramarginal postcentral precuneus posterior/isthmus cingulate paracentral superior frontal caudal anterior cingulate |
|  |  | 2 | 5923.45 | 0.0002 | 4.366 | -46.5 | -31.3 | -5.9 | middletemporal | superior temporal insula |
|  |  | 3 | 2641.27 | 0.0016 | 2.72 | -8.5 | 55.6 | -2.2 | medialorbitofrontal | superior frontal |
|  |  | 4 | 2484.67 | 0.0032 | 3.352 | -34.4 | 12.6 | 24.7 | caudalmiddlefrontal | rostral middle frontal pars opercularis precentral |
|  |  | 5 | 2020.95 | 0.02049 | 2.423 | -30.2 | -56.3 | -7.6 | lingual | fusiform inferior temporal |
|  | volume | 1 | 2270.88 | 0.0002 | 3.686 | -39.9 | -7.7 | -17.1 | superiortemporal | insula |
|  |  | 2 | 1259.01 | 0.00659 | 4.598 | -28.2 | -48.2 | 61.8 | superiorparietal | paracentral precuneus |
|  |  | 3 | 1222.53 | 0.00818 | 3.41 | -48.5 | -35.1 | -8.0 | middletemporal | superior temporal |
| right | area | 1 | 11814.59 | 0.0002 | 4.721 | 14.4 | -66.3 | 54.7 | superiorparietal | inferior parietal supramarginal postcentral precuneus parancentral superior frontal caudal anterior cingulate |
|  |  | 2 | 6122.35 | 0.0002 | 4.16 | 37.6 | 21.3 | 23.8 | rostralmiddlefrontal | caudal middle frontal pars opercularis pre- and postcentral |
|  |  | 3 | 4074.57 | 0.0002 | 4.005 | 47.1 | -21.2 | -6.7 | superiortemporal | insula middle temporal |
|  |  | 4 | 2738.67 | 0.0028 | 3.717 | 9.8 | 55.3 | -8.6 | medialorbitofrontal | superior frontal rostral anterior cingulate |
|  | volume | 1 | 6140.15 | 0.0002 | 4.001 | 37.9 | 1.7 | -19.2 | insula | precentral postcentral caudal middle frontal parts opercularis |
|  |  | 2 | 4038.12 | 0.0002 | 3.875 | 33.9 | -71.7 | 35.5 | inferiorparietal | superior parietal supramarginal |
|  |  | 3 | 1271.82 | 0.00459 | 4.836 | 9.9 | 51.8 | -3.0 | medialorbitofrontal | superior frontal rostral anterior cingulate |
|  |  | 4 | 972.17 | 0.03862 | 2.946 | 6.2 | -57.8 | 29.6 | precuneus | cuneus |
|  |  | 5 | 967.36 | 0.0394 | 4.636 | 45 | -24.8 | -7.3 | superiortemporal | middle temporal |

**CWP: cluster-wise significance (p-value). Max Vertex: -log(p) of most significant vertex within cluster and corresponding MNI coordinates (X, Y, Z). Columns on the right present regional locations of the most significant vertex in a cluster and of additional brain areas contained in the corresponding cluster (label obtained from Desikan Atlas).**

**Supplementary Table 2: Group comparisons of measures extracted over clusters listed in Supplementary Table 1.**

| **cluster** | **Dependent Variable** | **PD (mean ± SD)** | **HC (mean ± SD)** | **F(1,101)** | **p-value** | **Cohen's d** |
| --- | --- | --- | --- | --- | --- | --- |
| 1 | left area | 12303 ± 1184 | 13559 ± 1424 | 23.99 | <0.001 | 0.96 |
| 2 | left area | 6798 ± 556 | 7326 ± 646 | 20.28 | <0.001 | 0.88 |
| 3 | left area | 3235 ± 297 | 3421 ± 297 | 10.15 | 0.002 | 0.63 |
| 4 | left area | 2827 ± 323 | 3121 ± 427 | 15.64 | <0.001 | 0.78 |
| 5 | left area | 2576 ± 311 | 2780 ± 380 | 8.99 | 0.003 | 0.59 |
| 1 | left volume | 7445 ± 775 | 8068 ± 1001 | 14.27 | <0.001 | 0.70 |
| 2 | left volume | 3674 ± 573 | 4231 ± 792 | 18.20 | <0.001 | 0.81 |
| 3 | left volume | 4555 ± 626 | 5045 ± 606 | 22.56 | <0.001 | 0.80 |
| 1 | right area | 13630 ± 1185 | 15126 ± 1486 | 32.02 | <0.001 | 1.11 |
| 2 | right area | 6496 ± 660 | 7172 ± 863 | 19.92 | <0.001 | 0.88 |
| 3 | right area | 4640 ± 369 | 4969 ± 415 | 18.52 | <0.001 | 0.84 |
| 4 | right area | 3358 ± 305 | 3579 ± 357 | 11.50 | 0.001 | 0.67 |
| 1 | right volume | 17244 ± 1831 | 19184 ± 2350 | 24.85 | <0.001 | 0.92 |
| 2 | right volume | 12416 ± 1717 | 14310 ± 1672 | 36.82 | <0.001 | 1.12 |
| 3 | right volume | 4859 ± 529 | 5253 ± 625 | 15.35 | <0.001 | 0.68 |
| 4 | right volume | 2657 ± 367 | 2916 ± 485 | 9.56 | 0.003 | 0.60 |
| 5 | right volume | 3077 ± 393 | 3408 ± 511 | 15.89 | <0.001 | 0.73 |

Groups means and standard deviations (SD), statistical results of group comparisons, and effect sizes (Cohen’s d) are listed for cortical surface area and volume measures extracted from each cluster obtained in the main analysis (see Supplementary Table 1).

**Supplementary Table 3: Subcortical volume comparisons**

| **Region** | **Mean ± SD (PD)** | **Mean ± SD (HC)** | **F(1,101)** | **p-value** | **Cohen’s d** |
| --- | --- | --- | --- | --- | --- |
| **right accumbens** | **652 ± 114** | **709 ± 103** | **7.83** | **0.006*** | **0.52** |
| **right hippocampus** | **4527 ± 415** | **4755 ± 484** | **6.71** | **0.011*** | **0.5** |
| **left hippocampus** | **4384 ± 406** | **4593 ± 437** | **6.48** | **0.012*** | **0.5** |
| right pallidum | 2071 ± 227 | 2163 ± 193 | 4.95 | 0.028 | 0.44 |
| right putamen | 5337 ± 528 | 5551 ± 529 | 4.57 | 0.035 | 0.41 |
| left caudate | 3677 ± 421 | 3857 ± 476 | 4.36 | 0.039 | 0.4 |
| left putamen | 5275 ± 540 | 5484 ± 549 | 4.23 | 0.042 | 0.38 |
| left thalamus | 8468 ± 801 | 8823 ± 1021 | 4.16 | 0.044 | 0.39 |
| right caudate | 3767 ± 449 | 3947 ± 463 | 4.07 | 0.046 | 0.39 |
| right thalamus | 8294 ± 762 | 8534 ± 813 | 2.51 | 0.116 | 0.3 |
| left accumbens | 533 ± 109 | 564 ± 114 | 2.18 | 0.143 | 0.28 |
| right amygdala | 1951 ± 190 | 2002 ± 210 | 1.71 | 0.194 | 0.25 |
| left amygdala | 1817 ± 245 | 1851 ± 191 | 0.67 | 0.414 | 0.16 |
| left pallidum | 2178 ± 247 | 2211 ± 206 | 0.55 | 0.459 | 0.14 |

Groups means and standard deviations (SD) of subcortical volumes (in mm^3^), statistical results of group comparisons (PD vs. HC), and effect sizes (Cohen’s d) are listed. *: significant after FDR correction, considering all subcortical and WM volume measures analyzed.

**Supplementary Table 4: Regional white matter volume comparisons.**

| **Region** | **Mean ± SD (PD)** | **Mean ± SD (HC)** | **F(1,101)** | **p-value** | **Cohen’s d** |
| --- | --- | --- | --- | --- | --- |
| **lh_postcentral** | **8232 ± 923** | **9059 ± 1372** | **13.25** | **<0.001*** | **0.71** |
| **lh_precuneus** | **10715 ± 1574** | **11870 ± 1620** | **13.5** | **<0.001*** | **0.72** |
| **rh_inferiorparietal** | **12743 ± 1718** | **14397 ± 1776** | **23.07** | **<0.001*** | **0.95** |
| **rh_parsopercularis** | **3437 ± 602** | **3893 ± 571** | **15.66** | **<0.001*** | **0.78** |
| **lh_caudalmiddlefrontal** | **6960 ± 874** | **7555 ± 971** | **10.69** | **0.001*** | **0.64** |
| **lh_fusiform** | **7646 ± 945** | **8335 ± 1142** | **11.19** | **0.001*** | **0.66** |
| **lh_inferiorparietal** | **11056 ± 1537** | **12070 ± 1524** | **11.3** | **0.001*** | **0.66** |
| **lh_parsopercularis** | **3824 ± 678** | **4315 ± 826** | **10.8** | **0.001*** | **0.65** |
| **lh_superiorparietal** | **13794 ± 1717** | **15085 ± 2192** | **11.29** | **0.001*** | **0.66** |
| **lh_superiortemporal** | **8940 ± 1187** | **9711 ± 1161** | **11.22** | **0.001*** | **0.66** |
| **rh_precuneus** | **11233 ± 1460** | **12346 ± 1805** | **12** | **0.001*** | **0.68** |
| **rh_superiorparietal** | **13473 ± 1848** | **14712 ± 2064** | **10.81** | **0.001*** | **0.63** |
| **rh_frontalpole** | **399 ± 83** | **452 ± 86** | **10.21** | **0.002*** | **0.63** |
| **rh_caudalmiddlefrontal** | **6140 ± 1153** | **6827 ± 1182** | **9.19** | **0.003*** | **0.59** |
| **lh_middletemporal** | **6390 ± 857** | **6912 ± 959** | **8.52** | **0.004*** | **0.57** |
| **rh_precentral** | **14626 ± 1740** | **15697 ± 2040** | **8.8** | **0.004*** | **0.56** |
| **lh_posteriorcingulate** | **4856 ± 633** | **5271 ± 818** | **8.21** | **0.005*** | **0.57** |
| **rh_medialorbitofrontal** | **4234 ± 597** | **4557 ± 550** | **8.3** | **0.005*** | **0.56** |
| **rh_middletemporal** | **7208 ± 965** | **7724 ± 953** | **7.47** | **0.007*** | **0.54** |
| **lh_frontalpole** | **320 ± 64** | **354 ± 62** | **7.32** | **0.008*** | **0.54** |
| **rh_transversetemporal** | **615 ± 121** | **687 ± 148** | **7.21** | **0.008*** | **0.53** |
| **rh_caudalanteriorcingulate** | **2862 ± 423** | **3077 ± 421** | **6.68** | **0.011*** | **0.51** |
| **rh_superiortemporal** | **7558 ± 907** | **8026 ± 952** | **6.8** | **0.011*** | **0.5** |
| **rh_paracentral** | **5361 ± 708** | **5735 ± 773** | **6.6** | **0.012*** | **0.5** |
| **rh_postcentral** | **8194 ± 960** | **8805 ± 1427** | **6.48** | **0.012*** | **0.5** |
| **rh_supramarginal** | **9589 ± 1288** | **10359 ± 1820** | **6.06** | **0.016*** | **0.49** |
| **lh_precentral** | **14425 ± 1571** | **15251 ± 1978** | **5.86** | **0.017*** | **0.46** |
| **rh_entorhinal** | **971 ± 231** | **1086 ± 250** | **5.86** | **0.017*** | **0.48** |
| **rh_fusiform** | **7452 ± 920** | **7913 ± 1016** | **5.93** | **0.017*** | **0.48** |
| lh_supramarginal | 10174 ± 1677 | 10969 ± 1825 | 5.28 | 0.024 | 0.45 |
| lh_parstriangularis | 3449 ± 619 | 3713 ± 570 | 5.08 | 0.026 | 0.44 |
| lh_insula | 10726 ± 994 | 11176 ± 1027 | 5.12 | 0.026 | 0.45 |
| rh_temporalpole | 807 ± 139 | 868 ± 133 | 5.11 | 0.026 | 0.45 |
| rh_inferiortemporal | 7231 ± 1068 | 7681 ± 1046 | 4.78 | 0.031 | 0.43 |
| lh_caudalanteriorcingulate | 2869 ± 436 | 3081 ± 547 | 4.7 | 0.032 | 0.43 |
| lh_paracentral | 4413 ± 530 | 4648 ± 584 | 4.54 | 0.036 | 0.42 |
| rh_posteriorcingulate | 4627 ± 500 | 4847 ± 565 | 4.35 | 0.04 | 0.41 |
| lh_rostralmiddlefrontal | 14727 ± 2196 | 15605 ± 2153 | 4.24 | 0.042 | 0.4 |
| lh_entorhinal | 1091 ± 336 | 1223 ± 360 | 3.66 | 0.058 | 0.38 |
| lh_superiorfrontal | 20359 ± 2587 | 21378 ± 2991 | 3.54 | 0.063 | 0.36 |
| rh_superiorfrontal | 19994 ± 2565 | 21030 ± 3024 | 3.54 | 0.063 | 0.37 |
| lh_lateralorbitofrontal | 7793 ± 766 | 8094 ± 865 | 3.46 | 0.066 | 0.37 |
| rh_rostralanteriorcingulate | 2110 ± 324 | 2223 ± 299 | 3.4 | 0.068 | 0.36 |
| rh_parstriangularis | 3730 ± 611 | 3931 ± 587 | 2.92 | 0.091 | 0.34 |
| rh_insula | 10565 ± 1105 | 10913 ± 1125 | 2.51 | 0.117 | 0.31 |
| lh_rostralanteriorcingulate | 2945 ± 510 | 3091 ± 449 | 2.39 | 0.125 | 0.3 |
| lh_lateraloccipital | 12028 ± 1579 | 12502 ± 1740 | 2.19 | 0.142 | 0.29 |
| rh_bankssts | 3070 ± 488 | 3209 ± 489 | 2.08 | 0.152 | 0.28 |
| rh_parahippocampal | 1868 ± 237 | 1927 ± 228 | 1.72 | 0.193 | 0.25 |
| rh_lateralorbitofrontal | 7997 ± 1036 | 8273 ± 1152 | 1.66 | 0.2 | 0.25 |
| lh_medialorbitofrontal | 4409 ± 595 | 4596 ± 889 | 1.58 | 0.211 | 0.25 |
| lh_bankssts | 3212 ± 618 | 3368 ± 652 | 1.56 | 0.214 | 0.25 |
| lh_pericalcarine | 3851 ± 685 | 4040 ± 849 | 1.55 | 0.216 | 0.25 |
| lh_parsorbitalis | 1247 ± 235 | 1300 ± 200 | 1.49 | 0.225 | 0.24 |
| lh_parahippocampal | 1832 ± 279 | 1889 ± 216 | 1.44 | 0.233 | 0.23 |
| rh_rostralmiddlefrontal | 15169 ± 2201 | 15610 ± 2111 | 1.08 | 0.301 | 0.2 |
| lh_transversetemporal | 884 ± 147 | 915 ± 167 | 0.99 | 0.321 | 0.2 |
| lh_cuneus | 3180 ± 482 | 3278 ± 550 | 0.94 | 0.333 | 0.19 |
| lh_lingual | 6534 ± 880 | 6711 ± 1019 | 0.91 | 0.343 | 0.19 |
| rh_lateraloccipital | 12523 ± 1637 | 12837 ± 1886 | 0.83 | 0.364 | 0.18 |
| rh_lingual | 7006 ± 1086 | 7218 ± 1277 | 0.82 | 0.366 | 0.18 |
| lh_inferiortemporal | 7757 ± 1182 | 7959 ± 1100 | 0.81 | 0.37 | 0.18 |
| rh_parsorbitalis | 1552 ± 301 | 1600 ± 256 | 0.79 | 0.375 | 0.17 |
| lh_isthmuscingulate | 4175 ± 571 | 4267 ± 516 | 0.74 | 0.391 | 0.17 |
| lh_temporalpole | 800 ± 143 | 818 ± 131 | 0.54 | 0.466 | 0.13 |
| rh_cuneus | 3304 ± 474 | 3376 ± 560 | 0.5 | 0.481 | 0.14 |
| rh_pericalcarine | 4138 ± 842 | 4077 ± 804 | 0.14 | 0.71 | -0.07 |
| rh_isthmuscingulate | 3701 ± 481 | 3716 ± 419 | 0.03 | 0.871 | 0.03 |

Groups means and standard deviations (SD) of regional white matter (WM) volumes (in mm^3^), statistical results of group comparisons, and effect sizes (Cohen’s d) are listed. lh: left hemisphere, rh: right hemisphere. *: significant after FDR correction, considering all subcortical and WM volume measures analyzed.

***Pedophilic sexual orientation (PSO) related brain morphology (interpretational purpose)***

We found a significant effect of pedophilic sexual orientation (PSO) on some brain imaging outcomes in PD patients. To rule out a potential confound by PSO, we further explored this effect on vertex level by comparing brain imaging measures between g-PSO (PD patients attracted to girls) and b-PSO (PD patients attracted to boys or boys and girls) using same statistical models as described for the main analysis, but group was defined as g-PSO vs. b-PSO. We further tested for the effect of a 3-level factor (coding for HC, g-PSO, b-PSO) on extracted cortical measures in the combined cohort, and conducted pairwise group comparisons (SPSS).

The vertex-wise analysis comparing g-PSO and b-PSO revealed lower volume of b-PSO in right middle temporal cortex, right medial orbitofrontal, and left medial superior frontal cortex (Supplementary Figure 2). The temporal cluster obtained overlapped with the temporal cluster obtained in main analysis. Using extracted volumes of the temporal cluster obtained in the main analysis, the effect of the 3-level factor of PSO on cluster volume was significant (F(100,2)=10.75, p<0.001). However, pairwise group comparisons showed that HC had larger temporal volumes (3408 ± 510) than both g-PSO (3146 ± 379, p=0.003) and b-PSO (2830 ± 358, p<0.001), whereby b-PSO showed lower volume than g-PSO (p=0.028). The effect of the 3-level factor of PSO on right caudal ACC WM volume was significant (F(100,2)=5.84, p=0.004). Pairwise group comparisons showed that HC had larger caudal ACC WM volumes (3076 ± 421) than b-PSO (2620 ± 147, p=0.001) but not significantly larger than g-PSO (2929 ± 451, p=0.096). b-PSO showed lower caudal ACC WM volumes than g-PSO (p=0.032). These analyses indicated that a confound by PSO is unlikely, but b-PSO may have an additive effect on right temporal volume (and potentially caudal ACC WM volume), unrelated to PD *per se*.

**Supplementary Figure 2: Pedophilic sexual orientation (PSO)**


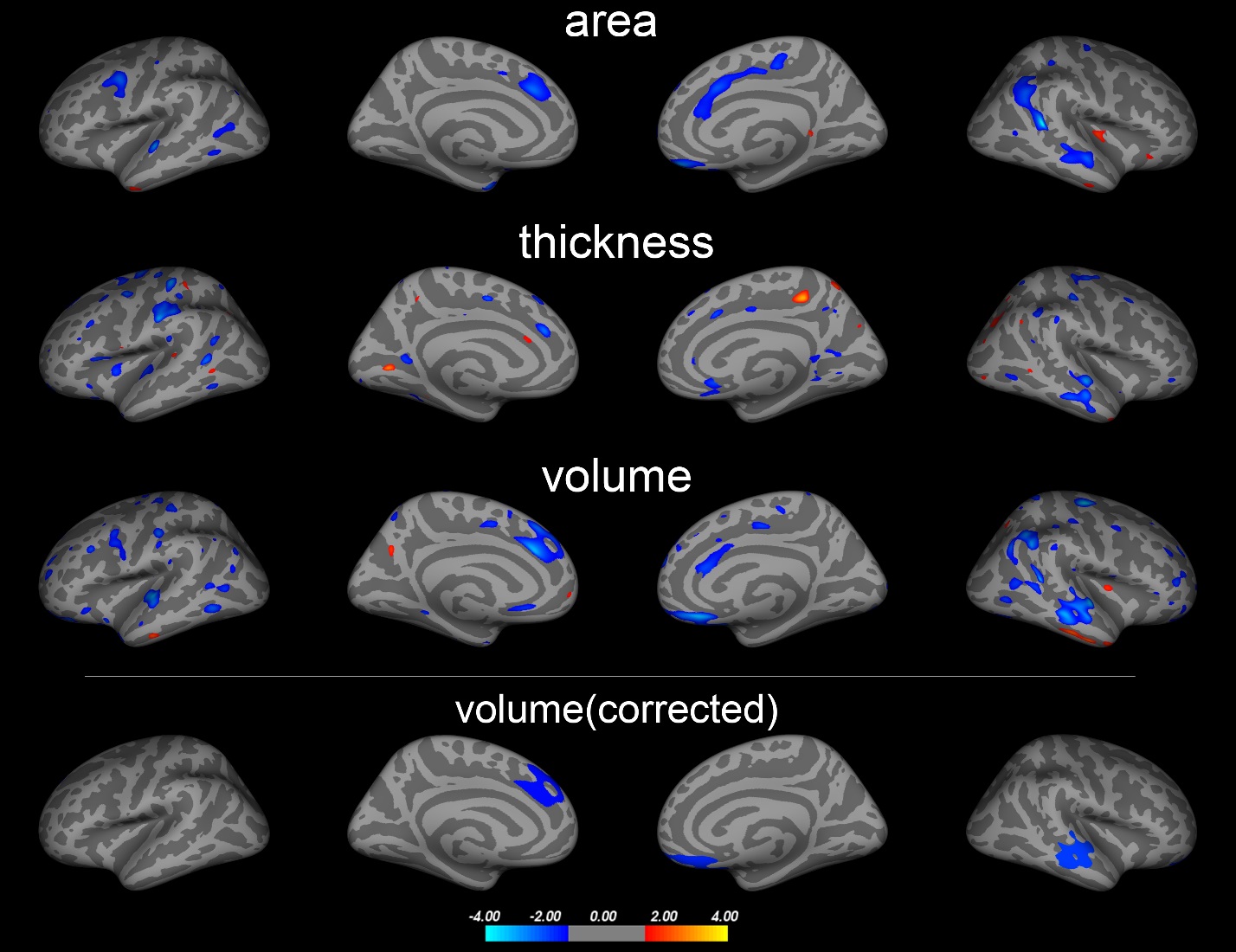


Main effect of pedophilic sexual orientation (PSO) in PD on cortical structures (three top rows: uncorrected threshold p<=0.05). Statistical significance is displayed on a log(p)-scale where positive values (warm colours) represent g-PSO < b-PSO, and negative values (cold colors) represent b-PSO < g-PSO. After Monte Carlo correction, significant volume differences where observed (bottom row).


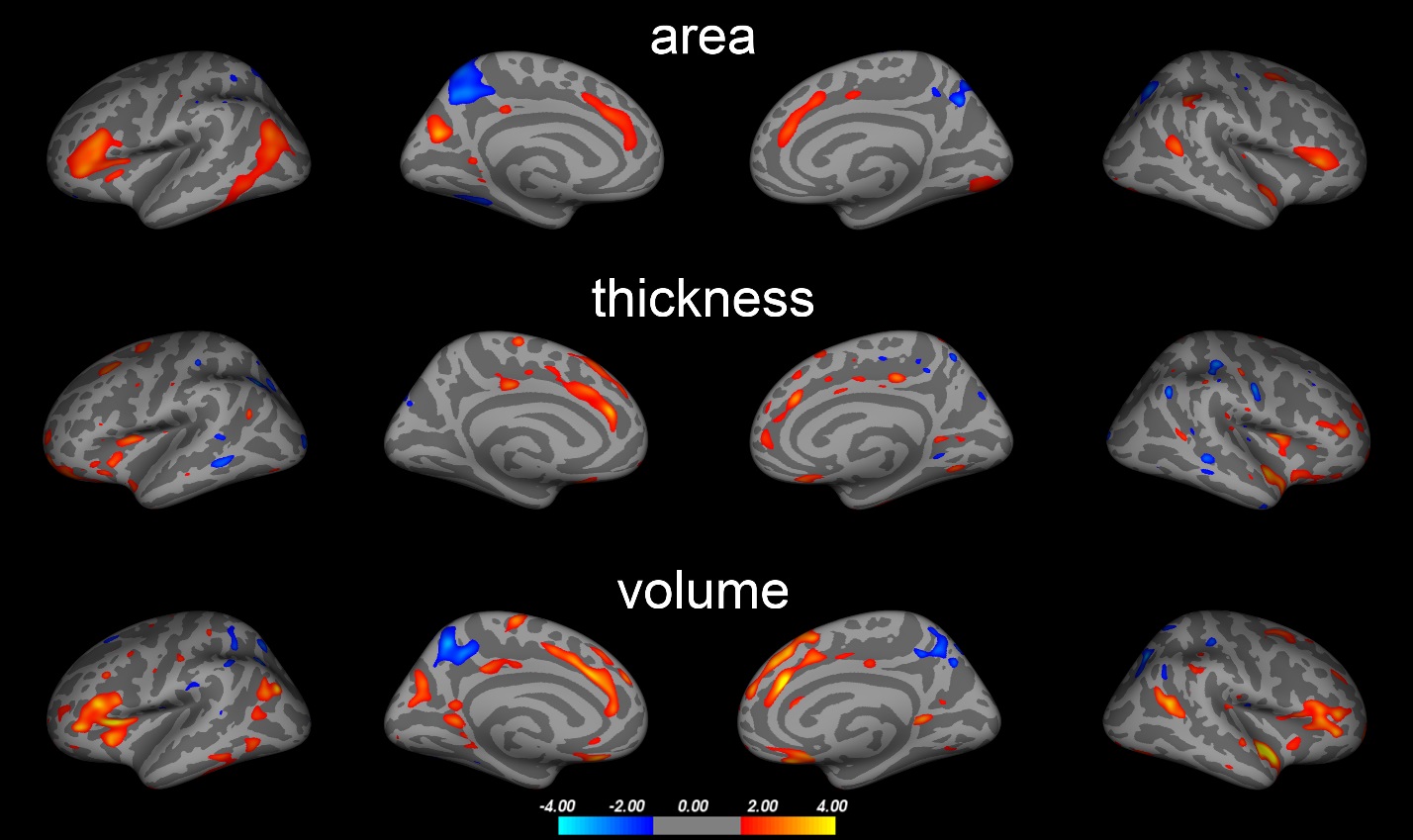


**Supplementary Figure 3: 2D:4D correlations (uncorrected).** Correlations between 2D:4D ratios and cortical surface area, thickness, and volume in PD. Colored areas represent brain regions in which significant correlations were observed (uncorrected threshold at p<0.05; see Figure 2 for corrected results). Statistical significance is displayed on a -log(p) scale. Cold colors represent negative and warm colors positive correlations.

**Supplementary Figure 4: Pial surface vs. WM surface area comparison**

*
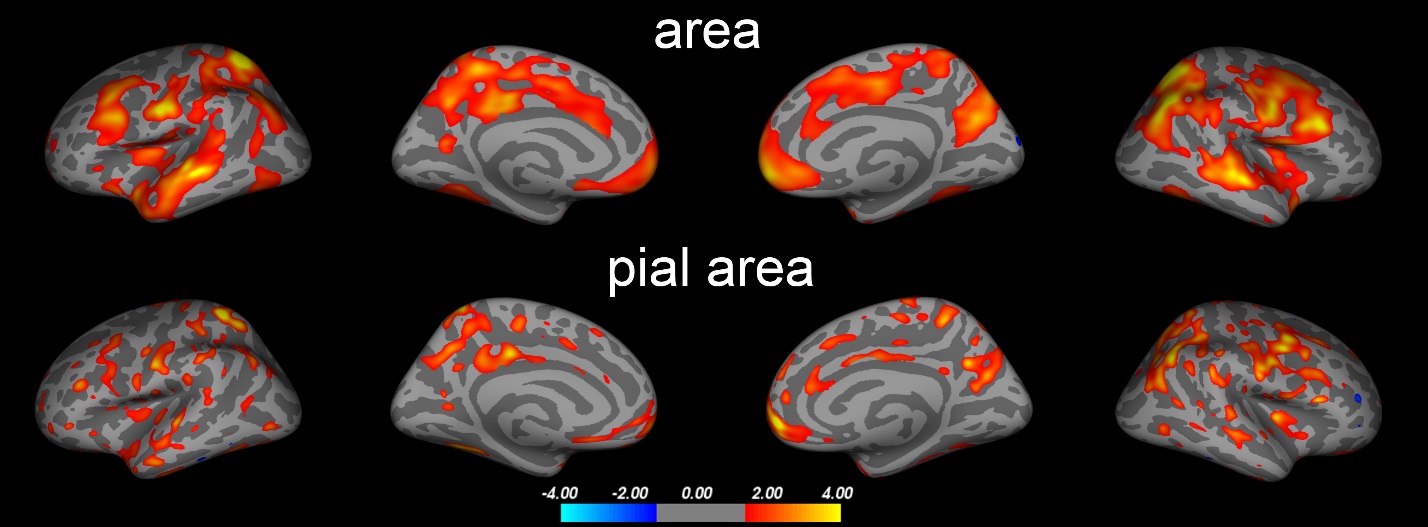
*

Group differences in WM surface (grey/white matter boundary, top; main analysis) in comparison with group differences obtained in pial surface area (grey matter/CSF boundary, bottom) displayed at uncorrected threshold p<0.05. Statistical significance is displayed on a log(p)-scale. Positive values (warm colours) represent PD < HC patterns.

**Supplementary Table 5: Sensitivity tests on main clusters**

| cluster | Dependent Variable | main | IQ | ICV |
| --- | --- | --- | --- | --- |
| 1 | left area | <0.001 | .001 | .000 |
| 2 | left area | <0.001 | .004 | .001 |
| 3 | left area | 0.002 | .070 | .072 |
| 4 | left area | <0.001 | .004 | .005 |
| 5 | left area | 0.003 | .077 | .063 |
| 1 | right area | <0.001 | .000 | .000 |
| 2 | right area | <0.001 | .004 | .001 |
| 3 | right area | <0.001 | .003 | .001 |
| 4 | right area | 0.001 | .095 | .046 |
| 1 | left volume | <0.001 | .015 | .012 |
| 2 | left volume | <0.001 | .001 | .001 |
| 3 | left volume | <0.001 | .001 | .000 |
| 1 | right volume | <0.001 | .001 | .000 |
| 2 | right volume | <0.001 | .000 | .000 |
| 3 | right volume | <0.001 | .025 | .007 |
| 4 | right volume | 0.003 | .033 | .045 |
| 5 | right volume | <0.001 | .000 | .004 |

Statistical significance (p-values) for group comparisons obtained when correcting for demographic and clinical variables in sensitivity tests. Only those control variables for which the significance of group differences changed are listed. P-values that increased to p > 0.05 are highlighted in red. Results obtained when not correcting for the test variable are listed for comparison (main; same as given in Supplementary Table 2). Note, IQ and ICV are likely related to PD phenotype. Correcting for those variables may disguise effects of interest. Thus, results of these analyses should be treated with caution.

**Supplementary Table 6: Sensitivity tests on subcortical and WM volumes**

| **Dependent Variable** | **main** | **Sexual offending** | **Antidepressant use** | **IQ** | **Social Anxiety** | **ICV** | **Hyper-sexuality** | **Excluding b-PSO** |
| --- | --- | --- | --- | --- | --- | --- | --- | --- |
| left hippocampus | 0.012 | 0.014 | 0.026 | .104 | .025 | 0.222 | .104 | .032 |
| right hippocampus | 0.011 | 0.008 | 0.013 | .154 | .031 | 0.146 | .232 | .006 |
| right accumbens-area | 0.006 | 0.015 | 0.004 | .052 | .004 | 0.068 | .022 | .022 |
| wm_lh_postcentral | .000 | 0.003 | 0.002 | .051 | .001 | 0.019 | .005 | .004 |
| wm_lh_precuneus | .000 | .000 | 0.001 | .020 | .001 | 0.012 | .002 | .001 |
| wm_rh_inferiorparietal | .000 | .000 | .000 | .001 | .000 | .000 | .000 | .000 |
| wm_rh_parsopercularis | .000 | 0.001 | .000 | .012 | .000 | 0.004 | .003 | .000 |
| wm_lh_caudalmiddlefrontal | .000 | 0.004 | 0.006 | .049 | .000 | 0.055 | .007 | .013 |
| wm_lh_fusiform | 0.001 | 0.001 | 0.006 | .080 | .014 | 0.047 | .000 | .005 |
| wm_lh_inferiorparietal | 0.001 | 0.005 | 0.005 | .066 | .008 | 0.031 | .000 | .006 |
| wm_lh_parsopercularis | 0.001 | 0.004 | 0.002 | .067 | .017 | 0.026 | .018 | .001 |
| wm_lh_superiorparietal | 0.001 | 0.001 | 0.003 | .068 | .001 | 0.025 | .006 | .004 |
| wm_rh_frontalpole | 0.002 | 0.005 | 0.001 | .153 | .005 | 0.021 | .000 | .004 |
| wm_rh_caudalmiddlefrontal | 0.003 | 0.02 | 0.017 | .080 | .005 | 0.085 | .101 | .004 |
| wm_lh_middletemporal | 0.004 | 0.024 | 0.008 | .077 | .033 | 0.117 | .035 | .027 |
| wm_lh_frontalpole | 0.008 | 0.012 | 0.009 | .175 | .002 | 0.076 | .045 | .011 |
| wm_rh_transversetemporal | 0.008 | 0.009 | 0.021 | .192 | .037 | 0.098 | .064 | .064 |
| wm_rh_caudalanteriorcingulate | 0.011 | 0.123 | 0.046 | .120 | .030 | 0.148 | .028 | .113 |
| wm_rh_superiortemporal | 0.011 | 0.009 | 0.017 | .203 | .044 | 0.254 | .046 | .019 |
| wm_rh_paracentral | 0.012 | 0.064 | 0.046 | .119 | .015 | 0.13 | .010 | .054 |
| wm_rh_postcentral | 0.012 | 0.018 | 0.034 | .316 | .010 | 0.37 | .031 | .033 |
| wm_rh_supramarginal | 0.016 | 0.018 | 0.026 | .275 | .061 | 0.351 | .049 | .035 |
| wm_lh_precentral | 0.017 | 0.018 | 0.054 | .232 | .009 | 0.412 | .049 | .048 |
| wm_rh_entorhinal | 0.017 | 0.007 | 0.067 | .209 | .025 | 0.108 | .056 | .026 |
| wm_rh_fusiform | 0.017 | 0.011 | 0.04 | .505 | .067 | 0.415 | .006 | .044 |
| wm_lh_superiortemporal | 0.001 | 0.001 | 0.002 | .175 | .008 | 0.038 | .015 | .006 |
| wm_rh_precuneus | 0.001 | 0.001 | 0.001 | .029 | .001 | 0.023 | .011 | .002 |
| wm_rh_precentral | 0.004 | 0.002 | 0.014 | .125 | .003 | 0.091 | .021 | .016 |
| wm_lh_posteriorcingulate | 0.005 | 0.057 | 0.008 | .147 | .005 | 0.147 | .068 | .007 |
| wm_rh_medialorbitofrontal | 0.005 | 0.01 | 0.023 | .415 | .007 | 0.176 | .016 | .027 |
| wm_rh_middletemporal | 0.007 | 0.035 | 0.016 | .213 | .050 | 0.304 | .023 | .024 |
| wm_rh_superiorparietal | 0.001 | .000 | 0.008 | .085 | .003 | 0.032 | .008 | .003 |

P-values for group comparisons obtained when correcting for demographic and clinical variables in sensitivity tests. Only those control variables for which the significance of group differences changed are listed. P-values that increased to p > 0.05 are highlighted in red. Results obtained when not correcting for the test variable are listed for comparison (main; same as given in Supplementary Table 4). Note, the listed variables are likely related to PD phenotype. Thus, results obtained when correction for these variables should be treated with caution.

**Supplementary Table 7: Multivariate regression model testing for the effects of 2D4D, IQ, and other clinical variables on LV-scores (global morphometric PD abnormality expression score)**

| Combined group | Estimate (beta) | SE | t | p-value |
| --- | --- | --- | --- | --- |
| (Intercept) | 24.917 | 11.311 | 2.203 | 0.030 |
| Age | 0.050 | 0.055 | 0.896 | 0.373 |
| Group | 3.381 | 2.086 | 1.621 | 0.108 |
| 2D:4D | -2.795 | 10.959 | -0.255 | 0.799 |
| Sexual offender status | -0.339 | 2.128 | -0.159 | 0.874 |
| Social anxiety disorder | -0.799 | 1.940 | -0.412 | 0.681 |
| Hypersexual behavior | -0.443 | 1.883 | -0.235 | 0.814 |
| **IQ** | **-0.231** | **0.056** | **-4.153** | **0.000** |
| PD |  |  |  |  |
| (Intercept) | 20.683 | 15.887 | 1.302 | 0.200 |
| Age | 0.075 | 0.074 | 1.008 | 0.320 |
| 2D:4D | 2.611 | 15.461 | 0.169 | 0.867 |
| Sexual offender status | -0.531 | 2.052 | -0.259 | 0.797 |
| Social anxiety disorder | -0.482 | 1.854 | -0.260 | 0.796 |
| Hypersexual behavior | 0.167 | 1.870 | 0.089 | 0.929 |
| **IQ** | **-0.202** | **0.069** | **-2.915** | **0.006** |
| Antidepressant use | 0.367 | 2.026 | 0.181 | 0.857 |
| PSO | -2.647 | 2.295 | -1.154 | 0.256 |
| HC |  |  |  |  |
| (Intercept) | 31.540 | 16.393 | 1.924 | 0.060 |
| Age | 0.036 | 0.083 | 0.431 | 0.669 |
| 2D:4D | -0.852 | 16.288 | -0.052 | 0.958 |
| **IQ** | **-0.300** | **0.094** | **-3.200** | **0.002** |

Results of three multivariate regression analyses are presented. Analyses were performed in the combined cohort (top), in PD patients (middle) and HC (bottom) only. Beta, t, and p-values are given for each predictor variable. SE: standard error.

**
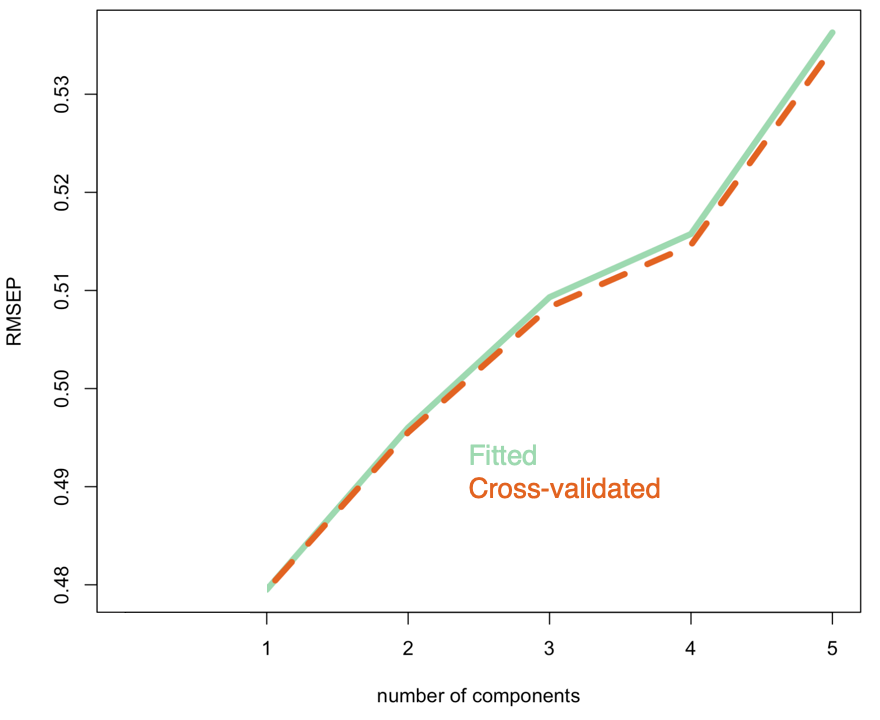
**

**Supplementary Figure 5: PLS classification analysis.** Root Mean Squared Prediction Error (RMSEP) as a function of the number of Partial Least Squares (PLS) components reveals the lowest RMSEP using one component.

**
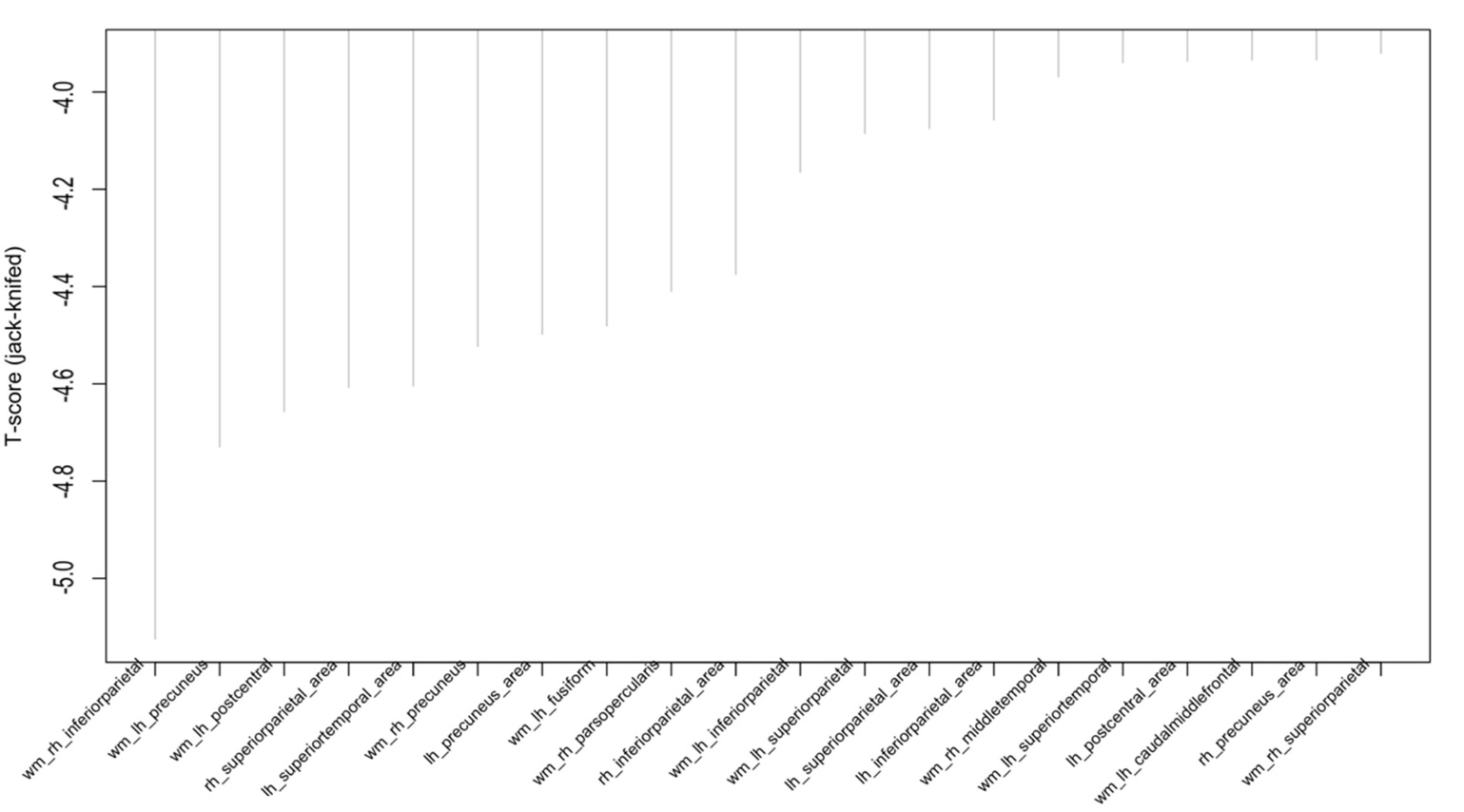
**

**Supplementary Figure 6: Top 20 features in PD vs. HC classification.** Jackknifed (aggregated estimate) t statistics for the 20 most important features used in Partial Least Squares classification with a 1-LV solution. area: cortical surface area. wm: white matter volume. lh: left hemisphere, rh: right hemisphere. Regional labels correspond to the Desikan atlas.

**Supplementary Table 8: Partial correlation between 2D:4D and white matter vol. in PD**

| **Partial Correlation** | **2D:4D** | |
| --- | --- | --- |
| **Region** | **r** | **p-value** |
| **wm_rh_caudalanteriorcingulate** | **0.382** | **0.007*** |
| **wm_rh_superiorparietal** | **-0.354** | **0.014*** |
| **wm_lh_superiorparietal** | **-0.331** | **0.022*** |
| wm_rh_frontalpole | 0.227 | 0.121 |
| wm_lh_middletemporal | 0.215 | 0.143 |
| wm_lh_parsopercularis | 0.208 | 0.155 |
| wm_rh_precentral | -0.171 | 0.246 |
| wm_lh_fusiform | -0.169 | 0.250 |
| wm_rh_postcentral | -0.156 | 0.289 |
| wm_lh_precuneus | -0.147 | 0.319 |
| wm_rh_supramarginal | 0.124 | 0.400 |
| wm_lh_precentral | -0.113 | 0.444 |
| left hippocampus | 0.096 | 0.515 |
| wm_rh_superiortemporal | 0.096 | 0.515 |
| wm_rh_fusiform | -0.091 | 0.541 |
| wm_rh_transversetemporal | 0.082 | 0.578 |
| wm_lh_posteriorcingulate | 0.080 | 0.588 |
| wm_lh_superiortemporal | 0.073 | 0.624 |
| right hippocampus | 0.069 | 0.643 |
| wm_rh_medialorbitofrontal | 0.067 | 0.650 |
| wm_lh_postcentral | -0.066 | 0.657 |
| wm_rh_precuneus | -0.053 | 0.722 |
| wm_rh_caudalmiddlefrontal | 0.045 | 0.761 |
| wm_rh_parsopercularis | 0.044 | 0.767 |
| wm_rh_middletemporal | 0.041 | 0.783 |
| wm_rh_inferiorparietal | -0.039 | 0.791 |
| wm_lh_frontalpole | 0.034 | 0.820 |
| wm_rh_entorhinal | -0.032 | 0.829 |
| wm_rh_paracentral | 0.018 | 0.904 |
| wm_lh_caudalmiddlefrontal | 0.018 | 0.906 |
| wm_lh_inferiorparietal | -0.009 | 0.949 |
| right accumbens-area | -0.005 | 0.973 |

Correlation coefficient (r) and significance (p-value) of partial correlations (correcting for age) between regional white matter volumes and 2D:4D in PD (n=49). Regions that showed significant group differences were analyzed. * significant at p<0.05, not FDR corrected.

**References:**

1. Landgren V, Malki K, Bottai M, Arver S, Rahm C. Effect of Gonadotropin-Releasing Hormone Antagonist on Risk of Committing Child Sexual Abuse in Men With Pedophilic Disorder: A Randomized Clinical Trial. *JAMA psychiatry.* Apr 29 2020.

2. Dale AM, Fischl B, Sereno MI. Cortical surface-based analysis. I. Segmentation and surface reconstruction. *NeuroImage.* Feb 1999;9(2):179-194.

3. Fischl B, Sereno MI, Dale AM. Cortical surface-based analysis. II: Inflation, flattening, and a surface-based coordinate system. *NeuroImage.* Feb 1999;9(2):195-207.

4. Fischl B, Dale AM. Measuring the thickness of the human cerebral cortex from magnetic resonance images. *Proceedings of the National Academy of Sciences of the United States of America.* Sep 26 2000;97(20):11050-11055.

5. Fischl B, van der Kouwe A, Destrieux C, et al. Automatically parcellating the human cerebral cortex. *Cerebral cortex (New York, N.Y. : 1991).* Jan 2004;14(1):11-22.

6. Fischl B, Salat DH, van der Kouwe AJ, et al. Sequence-independent segmentation of magnetic resonance images. *NeuroImage.* 2004;23 Suppl 1:S69-84.

7. Desikan RS, Segonne F, Fischl B, et al. An automated labeling system for subdividing the human cerebral cortex on MRI scans into gyral based regions of interest. *NeuroImage.* Jul 1 2006;31(3):968-980.

8. Hagler DJ, Jr., Saygin AP, Sereno MI. Smoothing and cluster thresholding for cortical surface-based group analysis of fMRI data. *NeuroImage.* Dec 2006;33(4):1093-1103.

9. Wold S, Ruhe A, Wold H, W. J. Dunn I. The Collinearity Problem in Linear Regression. The Partial Least Squares (PLS) Approach to Generalized Inverses. *SIAM Journal on Scientific and Statistical Computing.* 1984;5(3):735-743.

10. Khedher L, Ramírez J, Górriz JM, Brahim A, Segovia F. Early diagnosis of Alzheimer׳s disease based on partial least squares, principal component analysis and support vector machine using segmented MRI images. *Neurocomputing.* 2015/03/03/ 2015;151:139-150.

11. Hyatt CS, Owens MM, Crowe ML, Carter NT, Lynam DR, Miller JD. The quandary of covarying: A brief review and empirical examination of covariate use in structural neuroimaging studies on psychological variables. *NeuroImage.* Jan 15 2020;205:116225.
